# Testing Rare-Variant Association without Calling Genotypes Allows for Systematic Differences in Sequencing between Cases and Controls

**DOI:** 10.1371/journal.pgen.1006040

**Published:** 2016-05-06

**Authors:** Yi-Juan Hu, Peizhou Liao, H. Richard Johnston, Andrew S. Allen, Glen A. Satten

**Affiliations:** 1 Department of Biostatistics and Bioinformatics, Emory University, Atlanta, Georgia, United States of America; 2 Department of Biostatistics and Bioinformatics, Duke University, Durham, North Carolina, United States of America; 3 Centers for Disease Control and Prevention, Atlanta, Georgia, United States of America; University of California San Francisco, UNITED STATES

## Abstract

Next-generation sequencing of DNA provides an unprecedented opportunity to discover rare genetic variants associated with complex diseases and traits. However, the common practice of first calling underlying genotypes and then treating the called values as known is prone to false positive findings, especially when genotyping errors are systematically different between cases and controls. This happens whenever cases and controls are sequenced at different depths, on different platforms, or in different batches. In this article, we provide a likelihood-based approach to testing rare variant associations that directly models sequencing reads without calling genotypes. We consider the (weighted) burden test statistic, which is the (weighted) sum of the score statistic for assessing effects of individual variants on the trait of interest. Because variant locations are unknown, we develop a simple, computationally efficient screening algorithm to estimate the loci that are variants. Because our burden statistic may not have mean zero after screening, we develop a novel bootstrap procedure for assessing the significance of the burden statistic. We demonstrate through extensive simulation studies that the proposed tests are robust to a wide range of differential sequencing qualities between cases and controls, and are at least as powerful as the standard genotype calling approach when the latter controls type I error. An application to the UK10K data reveals novel rare variants in gene *BTBD18* associated with childhood onset obesity. The relevant software is freely available.

## Author Summary

In next-generation sequencing studies, there are typically systematic differences in sequencing qualities (e.g., depth) between cases and controls, because the entire studies are rarely sequenced in exactly the same way. It has long been appreciated that, in the presence of such differences, the standard genotype calling approach to detecting rare variant associations generally leads to excessive false positive findings. To deal with this, the current “state of the art” is to impose stringent quality control procedures that much of the data is eliminated. We present a method that allows analyzing data with a wide range of differential sequencing qualities between cases and controls. Our method is more powerful than the current practice and can accelerate the search for disease-causing mutations.

## Introduction

Recent technological advances in next-generation sequencing (NGS) have made it possible to conduct association studies on rare variants, which hold great potential to explain the missing heritability of complex traits and diseases [1]. However, it is prohibitively expensive to conduct high-depth, whole-genome sequencing (WGS) for large-scale association studies [2]. Therefore, many WGS studies have reduced the overall average depth to as low as 4–10× [3, 4]. Other studies have adopted whole-exome sequencing (WES), in which only the protein coding regions were sequenced but at high depth (e.g., ≥ 30×) [5, 6]; nevertheless, even though the *average* depth may be high, the large variability in capture efficiency may cause some genes or some regions within a gene to have much lower depth than the average [7].

The case-control design remains the most commonly used approach to studying rare variant associations. Due to the high cost of sequencing, many studies have focused sequencing effort on cases. Some studies sequenced cases at higher depth than controls by design, when the cases are unique and there is interest in identifying novel mutations [4]. Some studies even sampled only cases for sequencing and intended to compare them with publicly available NGS data on general populations such as the 1000 Genomes [3]. In both cases, the controls typically have systematically different sequencing qualities (e.g., depth and base-calling error rate) from the cases. Even when their *average* depths are similar, the *actual* depth could vary in individual regions across platforms, resulting in regions with differential depths in cases and controls by chance. This can easily occur when using different exome capture kits for cases and controls; if one kit can capture a certain exonic region better than the other, then there will be a systematic difference in read depth between cases and controls in this region.

The prevailing practice of analyzing NGS data for association with rare single-nucleotide variants (SNVs) is to first call underlying genotypes (e.g., using SAMtools [8] or GATK [9]), and then treat the called values as known in gene- or region-based tests such as the burden test [10, 11]. Genotype calling is difficult when read depth is low because minor allele reads are indistinguishable from sequencing errors. Genotype calling is especially challenging for rare SNVs, first because their locations cannot be easily inferred [12], and second because little information can be borrowed from other variants through linkage disequilibrium (LD) [3]. In case-control studies with differential sequencing qualities, the genotype calling process can introduce confounding that causes inflated type I error in downstream association tests [13]. Recall that confounding occurs when a variable is correlated with both the case-control status and the genotype. When read depths are different in cases and controls, the dependence of genotyping quality on the depth establishes the depth as a confounder. Likewise, the base-calling error rate has the same confounding effect as the depth. Even when read depths and error rates are comparable between cases and controls, differences in genotype calling algorithms or quality control (QC) filters (e.g., *phred* score cutoffs) can lead to differential genotyping errors that could also act as a confounder. For these reasons, publicly available NGS data have generally been under-utilized as controls for association studies. To reduce genotyping errors, one typically applies QC procedures to filter out SNVs at which many samples are covered by low depth of reads or called with low quality scores [5, 6]. The use of any reasonable QC procedure will remove a large number of variants, especially rare ones, and results in loss of important information.

An example is the UK10K Project [4], which sequenced cases at ∼ 60× and controls at ∼ 6×. In analysis of called genotypes, we obtained severely inflated type I error without QC (see Results). The UK10K Statistics Group adopted a series of QC procedures and controlled the type I error, but their QC removed 76.9% variants. Another example is the study of amyotrophic lateral sclerosis [6], which employed several sequencing platforms with unequal case-control ratios. Even when the average depth was as high as 144.6×, there were still at least 7.66% bases excluded from analysis due to depth less than 10×.

To avoid the confounding effect induced by calling genotypes, Derkach et al. [14] proposed to replace the genotypes in the standard score statistic by their expected values given observed read data, and developed a robust variance for the score statistic to account for differential variances of the expected genotypes in high- and low-depth samples. However, they still used called genotypes to determine SNV locations, which approach tends to yield more false positive SNVs among the low-depth group than the high-depth group and again cause confounding. To ensure accuracy of the called SNV locations, they resorted to stringent QC procedures, which would result in substantial information loss.

In this article, we provide a likelihood-based approach to testing rare variant associations that directly models sequencing reads without calling genotypes. We consider the (weighted) burden test statistic, which is the (weighted) sum of the score statistic for assessing effects of individual variants on the trait of interest. Our read-centric approach enables us to exploit genomic loci covered by low depth of reads and explicitly account for sequencing differences (i.e., read depth and error rate) between cases and controls.

**Fig 1:**
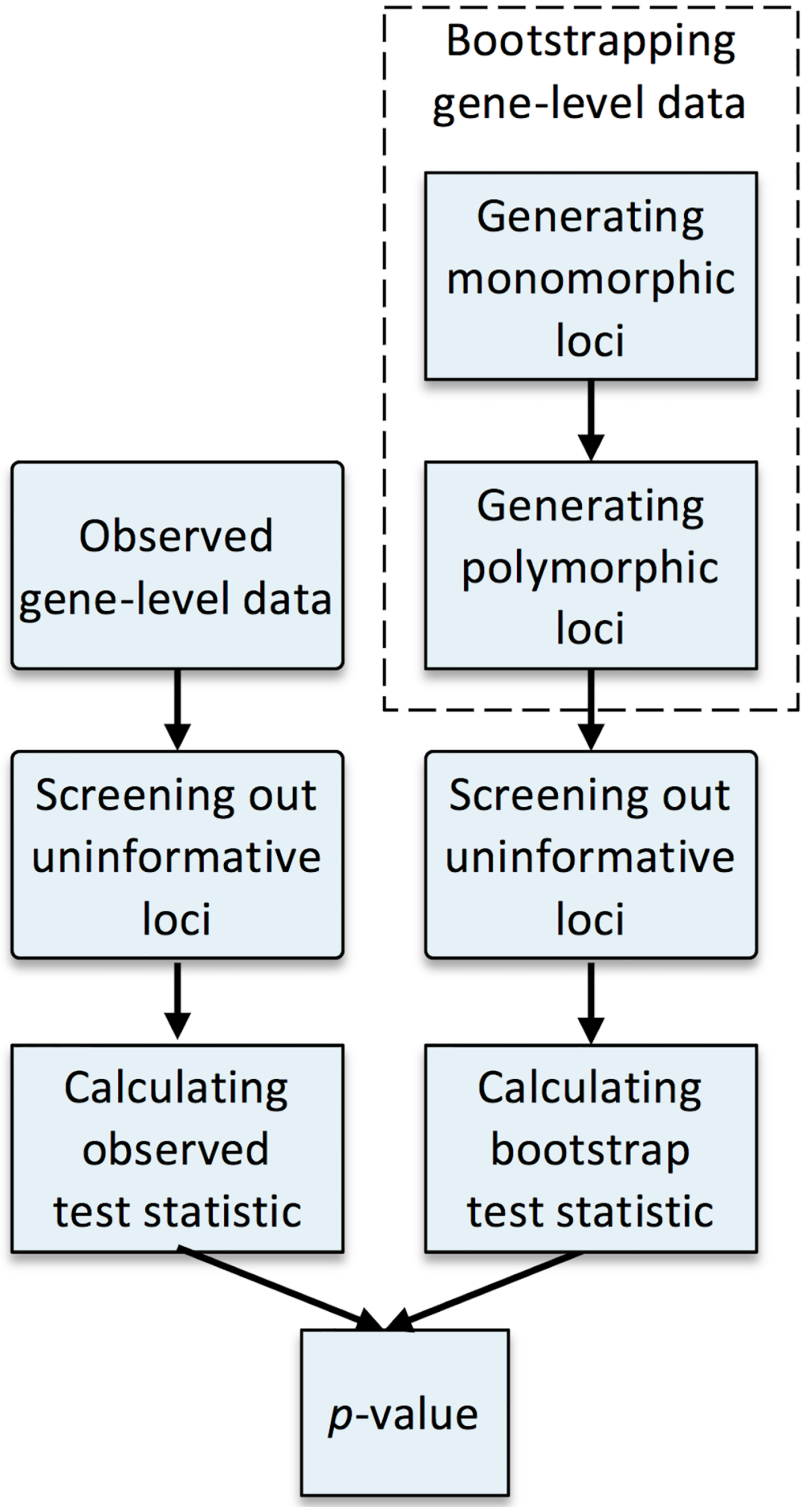
Flowchart of the proposed approach.

Full implementation of a read-centric approach requires solutions to a number of problems. Because SNV locations are unknown, we first develop a simple, computationally efficient screening algorithm to estimate their locations using read data alone. Because an imbalance in putative SNVs can arise due to differences in read depths and error rates between cases and controls, the burden statistic may not have mean zero even in the absence of association. Thus, we develop a novel bootstrap procedure for assessing the significance of the burden statistic. Specifically, in each bootstrap iteration, we propose to first generate a dataset with the same coverage patterns as the original data, but where the loci are all monomorphic. By comparing the false-positive SNVs found in the monomorphic dataset to the SNVs detected in the original data, we show how to estimate the number of true SNVs and the allele frequencies of the true SNVs in the original data. With this information, we can then generate a final bootstrap dataset in which the allele frequencies at true SNVs match those in the original data, but are identical in cases and controls. The entire procedure is repeated to generate multiple bootstrap datasets. Finally, we compare the burden statistic from the original data to those from the bootstrap datasets to assess significance. The complete flowchart is depicted in Fig 1. Our method can encompass all informative loci including singletons and doubletons if desired; additionally, we can down-weight or mask loci that are unlikely to be deleterious.

We showed through extensive simulation studies that our bootstrap tests are robust to a wide range of differential sequencing qualities between cases and controls, and are at least as powerful as the standard genotype calling approach when the latter controls type I error. We further applied the new methodology to a case-control data from the UK10K Project comparing children with severe early onset obesity to population-based controls. We identified a gene, *BTBD18*, that passes the exome-wide significance threshold and that is also a plausible candidate for childhood onset obesity.

## Materials and Methods

We first consider a single (bi-allelic) SNV. Let *G* be the genotype (coded as the number of minor alleles) at the variant site and let *D* be the disease status. We denote the genotype distribution under Hardy-Weinberg equilibrium (HWE) by *P*_*π*_(*G*), where *π* is the minor allele frequency (MAF). Note that the HWE assumption has a minimal effect for rare variants, as homozygotes of minor alleles are not expected. Instead of observing *G*, we observe the total number of reads mapped to the SNV and the number of reads carrying the minor allele, denoted by *T* and *R*, respectively. Similar to SAMtools, GATK, and seqEM [15], we assume that *R* given *T* and *G* follows a binomial distribution

$$P_{\epsilon }\left(R|T,G\right)=\left\{\begin{array}{cc} \text{Binomial}(T,\epsilon ) & \text{if}\;G=0\\ \text{Binomial}(T,0.5) & \text{if}\;G=1\\ \text{Binomial}(T,1-\epsilon ) & \text{if}\;G=2, \end{array}\right.$$ (1)

where *ϵ* is the probability that a read allele is different from the true allele and is referred to as the error rate. The “errors” here comprise both base-calling and alignment errors. We treat *ϵ* as a free parameter that is locus-specific and will be estimated from the read data [15].

### Test statistic

To account for case-control sampling, we adopt the retrospective likelihood with individual contribution

$$\text{Pr}\left(R_{i}|T_{i},D_{i}\right)=\sum_{g=0,1,2}\text{Pr}\left(R_{i}|T_{i},g,D_{i}\right)\text{Pr}\left(g|T_{i},D_{i}\right)=\sum_{g=0,1,2}\text{Pr}\left(R_{i}|T_{i},g\right)\text{Pr}(g|D_{i}),$$

where the second equation follows from two assumptions: first, the binomial distribution for read count data depends only on the underlying genotype, not on the disease status; second, the genotype distribution depends only on the disease status, not on the read depth. Thus, the likelihood based on *n* subjects takes the form

$$L_{\text{CC}}\left(\pi_{1},\pi_{0},\epsilon_{1},\epsilon_{0}\right)=\prod_{i\in D_{1}}\sum_{g=0,1,2}P_{\epsilon_{1}}\left(R_{i}|T_{i},g\right)P_{\pi_{1}}\left(g\right)\prod_{i\in D_{0}}\sum_{g=0,1,2}P_{\epsilon_{0}}\left(R_{i}|T_{i},g\right)P_{\pi_{0}}\left(g\right),$$ (2)

where $D_{1}$ and $D_{0}$ denote the sets of cases and controls, respectively, *π*_*d*_ denotes the allele frequency for *D* = *d*, and (*π*_1_, *ϵ*_1_) and (*π*_0_, *ϵ*_0_) are separate parameters for cases and controls. Note that in writing Eq (2) we assume that the depth *T* is independent of the genotype *G*. Also note that this formulation obviates the need to model other covariates (e.g., age and environmental exposures) as long as they are not confounders. The null hypothesis of the association test is *H*_0_: *π*_1_ = *π*_0_. We re-parameterize (*π*_1_, *π*_0_) in terms of (*α*, *β*) such that *π*_0_ = *e*^*α*^/(1 + *e*^*α*^) and *π*_1_ = *e*^*α*+*β*^/(1 + *e*^*α*+*β*^); then the null hypothesis is *H*_0_: *β* = 0. The score function for *β* under *H*_0_, as derived in S1 Text, can be written as

$$S=\sum_{i=1}^{n}\left(D_{i}-\frac{n_{1}}{n}\right){\tilde{G}}_{i},$$ (3)

where

$${\tilde{G}}_{i}=\frac{\sum_{g=0,1,2}gP_{{\tilde{\epsilon }}_{D_{i}}}\left(R_{i}|T_{i},g\right)P_{{\tilde{\pi }}_{0}}\left(g\right)}{\sum_{g=0,1,2}P_{{\tilde{\epsilon }}_{D_{i}}}\left(R_{i}|T_{i},g\right)P_{{\tilde{\pi }}_{0}}\left(g\right)},$$

*n*_1_ is the number of cases, and $\left({\tilde{\pi }}_{0},{\tilde{\epsilon }}_{1},{\tilde{\epsilon }}_{0}\right)$ are restricted maximum likelihood estimates (MLEs) under the null; these restricted MLEs can be obtained via the expectation-maximization (EM) algorithm described in S2 Text. ${\tilde{G}}_{i}$ can be interpreted as the posterior dosage of the minor allele (estimated under the null hypothesis); as the read depth increases, ${\tilde{G}}_{i}$ converges to the underlying genotype *G*_*i*_ and *S* reduces to the standard score statistic $\sum_{i=1}^{n}\left(D_{i}-n_{1}/n\right)G_{i}$. Finally, we construct the burden statistic *W* as a (weighted) sum of the score statistics at a set of SNVs in the gene of interest. The variance estimator *V* for *W* is calculated as the empirical variance of the efficient score functions [16]. When true SNVs are used, the test statistic $Z=W/\sqrt{V}$ is asymptotically normal with mean 0 and variance 1.

The score statistic of the Derkach test [14] has the same form as Eq (3), as it also uses the posterior dosage ${\tilde{G}}_{i}$. The only difference is that the Derkach test substitutes the genotype likelihood $P_{{\tilde{\epsilon }}_{D_{i}}}\left(R_{i}|T_{i},g\right)$ that is provided in the output of standard genotype calling packages [8, 9], which calculate error rates based on *phred* scores.

### Screening out uninformative loci

In reality, the locations of rare SNVs are not available without calling genotypes. In order to include the maximum set of variants in the burden test without calling genotypes, we develop a screening algorithm to screen every locus (i.e., base pair) in the genome and filter out only loci that are “uninformative” in the sense that they yield *S* = 0 and thus do not contribute to the test statistic. Specifically, we consider the likelihood $L_{S}\left(\pi ,\epsilon \right)=\prod_{i=1}^{n^{\prime }}\sum_{g=0,1,2}\;P_{\epsilon }\left(R_{i}|T_{i},g\right)P_{\pi }\left(g\right)$ which is based on a homogenous group (i.e., cases or controls only) of *n*′ subjects. Let $\tilde{\pi }$ be the MLE based on *L*_S_(*π*, *ϵ*)under the constraint that *π* ∈ [0, 1] and note that $\tilde{\pi }=0$ indicates no mutation in this group at this locus. Fortunately, we can easily determine whether $\tilde{\pi }=0$ without iteratively solving for $\tilde{\pi }$. By definition, $\tilde{\pi }$ also maximizes the profile likelihood *pl*(*π*) = max_*ϵ*_ log *L*_S_(*π*, *ϵ*). Because we have shown in S3 Text that *pl*(*π*) is a concave function of *π*, a negative derivative of *pl*(*π*) at *π* = 0 leads to $\tilde{\pi }=0$. At *π* = 0, the *ϵ* maximizing log *L*_S_(*π*, *ϵ*) can be easily determined because, in the absence of any minor alleles, all reads carrying the minor allele must be errors. Therefore, we check the sign of the derivative of *pl*(*π*) at *π* = 0 for cases and controls separately and screen out the loci at which both signs are negative. If $\tilde{\pi }=0$ in both cases and controls, then ${\tilde{\pi }}_{0}=0$ in the combined sample, where ${\tilde{\pi }}_{0}$ was defined in the text following expression Eq (3). From ${\tilde{\pi }}_{0}=0$, we have ${\tilde{G}}_{i}=0$ for all individuals and thus *S* = 0. This screening algorithm only involves evaluating simple (derivative) functions twice at each locus without any iteration, and is thus computationally extremely efficient.

### Bootstrap

Although most monomorphic loci are “uninformative” and will be screened out, there are exceptions. It is possible that a truly monomorphic locus has $\tilde{\pi }>0$ in one disease group or both, if by chance some individuals have more errors than expected. If a truly monomorphic locus has $\tilde{\pi }>0$ in the control group but $\tilde{\pi }=0$ in the case group, the score statistic *S* of this locus will have a negative mean. Such loci will accumulate over the gene when controls have systematically lower depth (or higher error rate) than cases, and then the expected value of the burden statistic *W* will be substantially biased below zero, even when allele frequencies are identical among cases and controls at true SNVs. Consequently, screening for SNVs in the presence of differential sequencing qualities between cases and controls will invalidate the asymptotic version of our test.

We thus propose a bootstrap procedure for assessing the significance of the observed test statistic *Z*. The idea is to generate bootstrap datasets that mimic the original data in terms of read depth and error rate, have the same number of truly monomorphic loci and true SNVs, but have no difference in allele frequencies among cases and controls. To this end, we condition on the observed depth *T* and simulate the minor-allele read count *R* using the estimated error rates ${\tilde{\epsilon }}_{1}$ and ${\tilde{\epsilon }}_{0}$ once the underlying genotype *G* is simulated. However, it is nontrivial to simulate *G*, because we do not know how many loci in the gene are true SNVs and what are allele frequencies at these SNVs. To obtain this information, we first form a “monomorphic” dataset by simulating *R* at every locus in the gene assuming that all *G*s are zero; thus, each read for the minor allele is an error that occurs with rate ${\tilde{\epsilon }}_{1}$ or ${\tilde{\epsilon }}_{0}$, depending on the disease status. This dataset should provide a good approximation to the truly monomorphic loci in the original data, as the proportion of true SNVs in the original data should be small. Let *M*_*s*_ be the number of loci that are screened in from the original data and let *F*_*s*_(*π*) be the cumulative distribution function (CDF) of estimated MAFs at the *M*_*s*_ loci. Let *M*_*m*_ and *F*_*m*_(*π*) be their counterparts in the monomorphic dataset. The CDF of allele frequencies at true SNVs, denoted by *F*_*p*_(*π*), is related to *F*_*s*_(*π*) and *F*_*m*_(*π*) through the equation

$$F_{s}\left(\pi \right)=\phi F_{m}\left(\pi \right)+\left(1-\phi \right)F_{p}\left(\pi \right),$$

where *ϕ* is the proportion of monomorphic loci among loci that are screened in. This equation expresses the fact that the distribution of observed (non-zero) allele frequencies *F*_*s*_(*π*) in the original data is a mixture of the distributions for allele frequencies of true SNVs *F*_*p*_(*π*) and artifactual SNVs *F*_*m*_(*π*) that actually correspond to monomorphic loci. We estimate *ϕ* by $\hat{\phi }=M_{m}/M_{s}$ and *F*_*p*_ by ${\hat{F}}_{p}(\pi )={\left(1-\hat{\phi }\right)}^{-1}\left\{{\hat{F}}_{s}(\pi )-\hat{\phi }{\hat{F}}_{m}(\pi )\right\}$, where ${\hat{F}}_{s}$ and ${\hat{F}}_{m}$ are empirical CDF estimators of *F*_*s*_(*π*) and *F*_*m*_(*π*) respectively. To ensure that ${\hat{F}}_{p}\left(\pi \right)$ is monotonically increasing, we refine ${\hat{F}}_{p}\left(\pi \right)$ by fitting an isotonic regression to data points of ${\left(1-\hat{\phi }\right)}^{-1}\{{\hat{F}}_{s}\left(\pi \right)-\hat{\phi }{\hat{F}}_{m}\left(\pi \right)\}$ evaluated at the pooled (*M*_*s*_ + *M*_*m*_) MAFs by the pooled-adjacent-violator algorithm (PAVA) [17]. After the largest value of MAF, we set ${\hat{F}}_{p}\left(\pi \right)=1$. Finally, starting from the monomorphic dataset, we select ${\hat{M}}_{p}=M_{s}-M_{m}$ loci to be SNVs, sample *π* from ${\hat{F}}_{p}$, and re-generate *G* and *R* at these SNVs to form a final bootstrap dataset. Note that, for a small *π*, we may need to resample *G* repeatedly until each truly polymorphic locus screens in. The bootstrap statistic is then calculated based on all the loci that were screened in from the final bootstrap dataset. The entire procedure is repeated to generate multiple bootstrap replicates.

Although bootstrap tests are computationally intensive in general, we can save considerable time by adopting a sequential stopping rule [18]. We stop after generating *L*_min_ bootstrap replicates, if these early replicates suggest a large *p*-value. When *L*_min_ = 5, the number of replicates at termination has a median of only 10 for a gene having no SNVs that affect the trait. We also use a closed sampling scheme, in which we restrict the total number of bootstrap replicates to be at most *K*_max_. If we stop when *L*_min_ bootstrap statistics exceed the observed *Z* and *K*_obs_ (≤*K*_max_) replicates have been collected, we set the *p*-value to *L*_min_/*K*_obs_. If we stop when *K*_max_ replicates are reached and only *L*_obs_ (<*L*_min_) values exceed *Z*, we set the *p*-value to (*L*_obs_ + 1)/(*K*_max_ + 1).

### Adjusted empirical Bayes estimator for error rate

The MLEs of error rates may not recover the true distribution of error rates, which is essential for generating valid bootstrap replicates. In particular, when the true error rates are very small (e.g., ∼ 0.02%), the MLEs tend to be over-dispersed. Therefore, we propose the following “adjusted” empirical Bayes (EB) estimator of the error rate to be used in bootstrap (instead of the MLE), which is calculated separately among cases and controls. We assume a prior beta distribution for error rates, i.e., *ϵ*_*j*_ ∼ *Beta*(*a*, *b*), where *j* = 1, …, *M*, *M* is the total number of loci in the gene, and *a* and *b* are hyperparameters that can be consistently estimated by the method of moments (see S4 Text). While the EB estimator is easily obtained (S4 Text), it is known that the distribution of EB estimators is over-shrunk [19]. Louis and Shen [19] proposed estimators that have good distribution, rank and expected value, but these are cumbersome to compute. We use a simplified version of the Louis and Shen estimator in which we first calculate the EB estimators but then replace the (ordered) EB estimators by (ordered) quantiles of the prior beta distribution evaluated using the method-of-moments estimators of *a* and *b*. Because the sample size *M* is typically on the order of a few hundred, *a* and *b* are accurately estimated, ensuring that the distribution of the adjusted EB estimates will closely resemble the prior (true) distribution of error rates.

### Read-based QC procedure

We have observed that a small proportion of read data (*R*, *T*) do not fit the binomial model (1). This may be due to genotype mosaicism (i.e., the presence of two or more populations of cells with different genotypes in one individual), experimental artifacts, sample contamination, or copy number variants. To detect data that do not fit the binomial model, for each individual at each locus that screens in, we calculated a likelihood-ratio-type statistic for the goodness of fit to the binomial model

$$Q=2\log\left\{{\left(R/T\right)}^{R}{\left(1-R/T\right)}^{T-R}/\max_{g=0,1,2}\left[e_{g}{\left(\epsilon \right)}^{R}{\left\{1-e_{g}\left(\epsilon \right)\right\}}^{T-R}\right]\right\},$$

where *e*_*g*_(*ϵ*) = *ϵ*, 0.5, and 1 − *ϵ* for *g* = 0, 1, and 2, respectively. Then, we mask an individual at a variant (by setting *T* and *R* to zero) if *Q* is greater than 10 and remove a variant altogether if more than 5 individuals are masked at that locus. We can also identify individuals with problematic data by checking for the presence of an excessive number of *Q*s greater than 10.

### Software

The proposed methods are implemented in the C/C++ program TASER, which is publicly available at http://web1.sph.emory.edu/users/yhu30/software.html.

## Results

### Simulation studies

We carried out extensive simulation studies to evaluate the performance of our proposed methods in realistic settings. We used the coalescent simulator cosi [20] to generate a base population of 100,000 European haplotypes with length 10 kb. We assumed that the 10 kb region corresponds to a gene with 3 exons that are separated by 2 introns, with introns being 3 times the length of exons. This setup gave us a total of 2,730 loci in exons, among which there are 44 SNVs with MAFs ≤ 0.05 in the base population. To generate individual genotypes, we sampled from the 100,000 haplotypes allowing recombination in introns (but not in exons). To generate disease outcomes, we considered a risk model that assumed equal attributable risk (AR) for each SNV: $\log\left\{P\left(D=1\right)/P\left(D=0\right)\right\}=\alpha +\sum_{j=1}^{m}G_{j}\log\left(1+\text{AR}/2\pi_{j}\right)$, where *m* is the total number of SNVs, *G*_*j*_ and *π*_*j*_ are the genotype and MAF of the *j*th SNV, and *α* was set to −3 to achieve a disease rate of ∼ 5%. This risk model implies that a more rare SNV has a stronger effect than a less rare SNV. The process was repeated until 500 cases and 500 controls were collected.

The sequencing reads *T* and *R* were generated to mimic real NGS data. We considered average read depths of 6×, 10×, and 30×, and average error rates of 0.02% and 0.016% (as observed in the UK10K cases and controls, respectively). While these very low error rates are characteristic of the newest Illumina platforms, we also considered average error rates of 1% and 0.5% that exist in historical NGS data [21]. We sampled the locus-specific error rate *ϵ* from a beta distribution that yields the pre-specified average rate. We sampled the individual depth *T* by a two-step strategy which first simulates the locus-specific mean depth *c* from a beta distribution (re-scaled to achieve the pre-specified average depth) and then simulates individual *T*’s from a negative-binomial distribution with mean *c*. The first step permits the accessibility of sequencing to depend on local nucleotides, and the second step allows for dispersion in the individual count data. For specific parameter values in these distributions, refer to S5 Text. Note that at each locus we sampled *ϵ* and *c* independently for cases and controls, mimicking the scenario in which the two groups have been sequenced as part of different studies (e.g., on different platforms), even when the average values are the same between the two groups. Finally, we sampled *R* given (*T*, *G*, *ϵ*) according to Eq (1).

We considered eight methods. First, we assumed that the 44 SNV locations were known and applied the asymptotic version of our method, the method using called genotypes that extends the multi-sample, single-locus genotyper seqEM [15] to allow for different error rates in cases and controls, the Derkach method using genotype dosages, and the method using true genotypes as a gold standard; we refer to them as New, CG, Dose, and True. Note that, to ensure fair comparisons, we used the error rates from our method in the implementation of the Derkach test, whose score statistic is then the same as our *S* in Eq (3). Thus, although Derkach *et al.* used a slightly different variance estimator for the score statistic, New and Dose are asymptotically equivalent. Next, we considered the more realistic case that the SNV locations are unknown. We applied our method including the screening and bootstrap procedures and refer to it as New-SB. While this method aims to maximize the set of true SNVs, it may also include a sizable number of monomorphic loci that can adversely affect the power of association testing. We thus explored a modification of New-SB, which adds a thresholding step that excludes loci with estimated MAFs <(2*n*)^−1^ and is referred to as New-STB. The threshold of (2*n*)^−1^ corresponds to the MAF of a singleton variant and can effectively remove the majority of monomorphic loci that accidentally pass the screening algorithm, although at a cost of potentially losing some true singletons. In addition, we applied the method of called genotypes and the Derkach method based on loci that were screened in and refer to them as CG-S and Dose-S.

We focused on the weighted burden test of SNVs with MAFs ≤ 5%, in which each SNV is inversely weighted by $\sqrt{\pi_{j}\left(1-\pi_{j}\right)}$[11]; results of the unweighted test are provided in S1 and S2 Tables. We first evaluated type I error of the burden test using the aforementioned methods and summarized the results in Table 1. All of the new methods (New, New-SB, New-STB) have correct type I error, regardless of how different the sequencing depths and error rates are between cases and controls. The genotype calling methods (CG, CG-S) generally have inflated type I error when the average depths are different between cases and controls. Their type I error tends to be inflated even when the average depths and error rates are the same but there are random differences in individual regions between cases and controls; the inflation in such a case is more noticeable for the unweighted test (S1 Table) than for the weighted test (Table 1), because the SNVs with higher MAFs contribute more to the inflation and they are down-weighted in the weighted test. Only when cases and controls have exactly the same sequencing feature at every locus, which can be achieved by sequencing cases and controls together, should the genotype calling methods have correct type I error. The Derkach approach worked well when the SNV locations are known, but its type I error rate can be as much as 88 times the nominal level when the locations are unknown. In Table 2, we give additional results on the behavior of our test statistics under the null hypothesis. We see that the test statistic in the presence of screening is negatively biased from zero when controls have lower average depth than cases, which confirms the need for our bootstrap test. We also see in Table 2 that, when the average error rate is high, the screening procedure screened in a large number of monomorphic loci, and that the thresholding procedure effectively removed many such loci. Finally, we see that the bootstrap procedure accurately estimated the number of truly polymorphic loci. S1 Fig shows that the MLEs of error rates are more dispersed than the true error rates (especially contain too many zeros when the average is 0.02%), the EB estimator imposed a strong shrinkage effect, and that our adjusted EB estimator accurately recovered the true distribution. S2 Fig shows that, when the average error rate is 1%, the monomorphic loci that were screened in are typically associated with small $\tilde{\pi }$’s, the majority of which are smaller than the threshold of (2*n*)^−1^.

**Table 1:** Type I error of the weighted burden test at the nominal significance level of 0.01.

				Known SNVs				Unknown SNVs			
*c*_1_	*c*_0_	*ϵ*_1_	*ϵ*_0_	New	CG	Dose	True	New-SB	New-STB	CG-S	Dose-S
6×	6×	0.02%	0.02%	0.010	0.011	0.009	0.009	0.011	0.011	0.011	0.009
30×	6×	0.02%	0.02%	0.010	0.055	0.009	0.009	0.010	0.010	0.033	0.161
30×	30×	0.02%	0.02%	0.009	0.010	0.009	0.010	0.010	0.010	0.010	0.010
30×	6×	0.02%	0.016%	0.011	0.061	0.010	0.011	0.009	0.009	0.029	0.143
10×	10×	1%	1%	0.008	0.010	0.008	0.009	0.011	0.008	0.012	0.011
30×	10×	1%	1%	0.008	0.037	0.008	0.010	0.011	0.008	0.358	0.878
30×	30×	1%	1%	0.010	0.011	0.011	0.010	0.011	0.009	0.012	0.012
30×	10×	1%	0.5%	0.011	0.024	0.011	0.010	0.011	0.008	0.379	0.702

*c*_1_ and *c*_0_ are average depths in cases and controls, respectively. *ϵ*_1_ and *ϵ*_0_ are average error rates in cases and controls, respectively. Each entry is based on 10,000 replicates.

**Table 2:** Other simulation results for the weighted burden test under the null hypothesis.

				New		New-SB			New-STB	
*c*_1_	*c*_0_	*ϵ*_1_	*ϵ*_0_	*Z*	*M*_*p*_	*Z*	*M*_*s*_	${\hat{M}}_{p}$	*Z*	*M*_*st*_
6×	6×	0.02%	0.02%	0.025	19.9	0.017	47.6	19.7	0.020	46.0
30×	6×	0.02%	0.02%	0.177	21.3	-1.443	34.9	21.3	-1.533	34.0
30×	30×	0.02%	0.02%	0.010	22.6	0.008	25.5	22.4	0.009	25.1
30×	6×	0.02%	0.016%	0.201	21.3	-1.423	34.7	21.3	-1.511	33.9
10×	10×	1%	1%	-0.013	20.5	-0.010	162.0	20.1	-0.008	62.9
30×	10×	1%	1%	0.027	21.4	-2.271	102.0	20.9	-1.150	38.6
30×	30×	1%	1%	0.004	22.4	0.001	55.4	22.1	0.001	28.0
30×	10×	1%	0.5%	0.018	21.6	-2.031	89.7	21.2	-0.849	36.0

*c*_1_ and *c*_0_ are average depths in cases and controls, respectively. *ϵ*_1_ and *ϵ*_0_ are average error rates in cases and controls, respectively. *Z* is the test statistic. *M*_*p*_ is the number of true SNVs. ${\hat{M}}_{p}$ is the estimated number of SNVs. *M*_*s*_ is the number of loci that were screened in. *M*_*st*_ is the number of loci that were screened in and passed the threshold. Each entry is based on 10,000 replicates.

**Fig 2:**
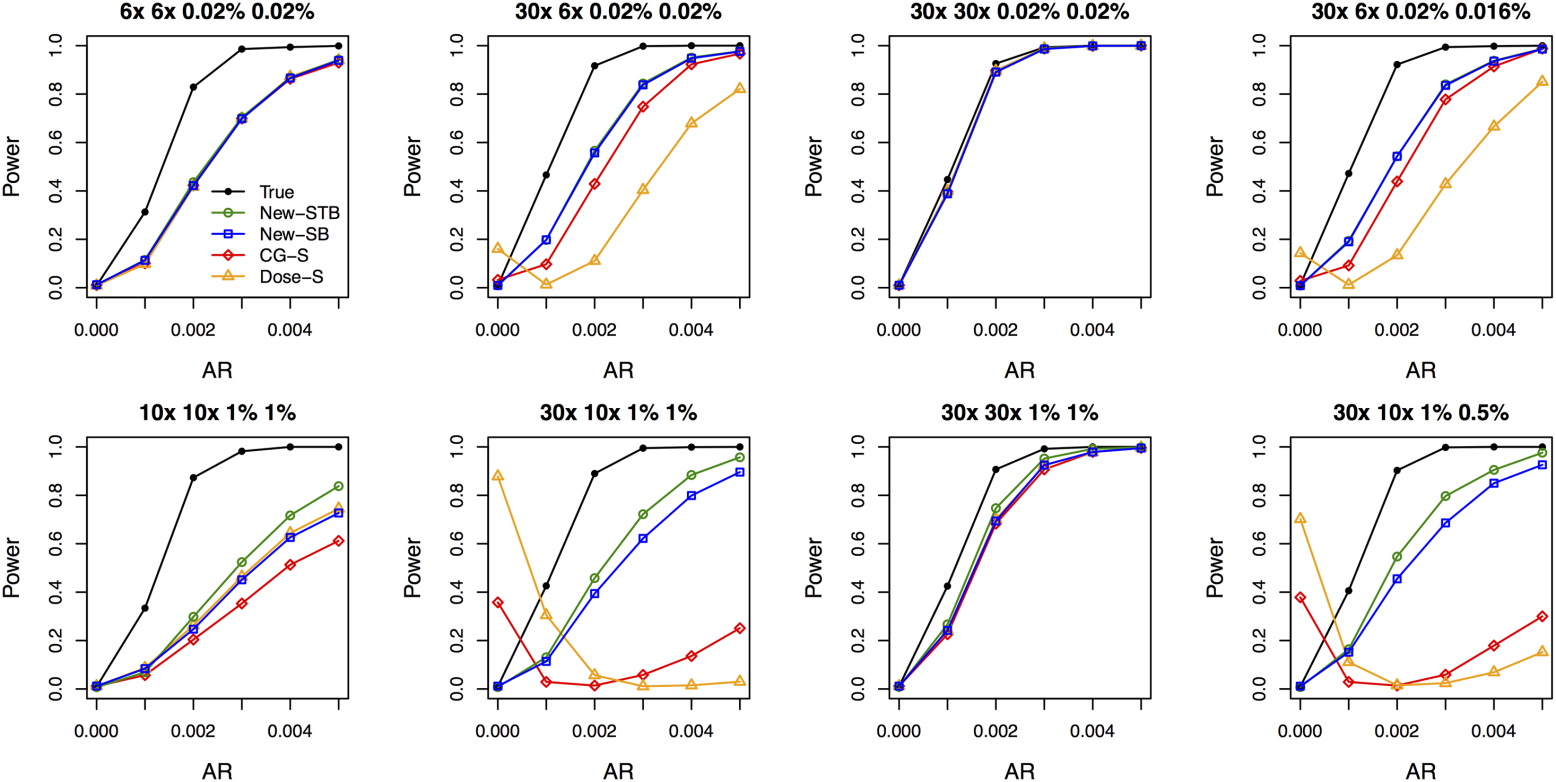
Power of the weighted burden test at the nominal significance level of 0.01. The title of each plot lists the average depths in cases and controls and then the average error rates in cases and controls. AR is the attributable risk per SNV. Each power estimate is based on 1,000 replicates.

Fig 2 contrasts the power of different methods. The thresholding strategy implemented in New-STB significantly improved the power of New-SB at error rate of ∼ 1% and performed as well as New-SB at ∼ 0.02%. In the presence of differential depths between cases and controls, the power of CG-S and Dose-S can even decrease as the effect size starts to increase from zero and both are substantially lower than the power of New-SB and New-STB at median and high effect sizes. In the presence of equal average depths and error rates, the power of CG-S and Dose-S are comparable to that of New-SB and New-STB at error rate of ∼ 0.02% and noticeably lower at ∼ 1% (even at high depth of ∼ 30×). Power curves pertaining to unweighted burden tests are displayed in S3 Fig, which shows similar patterns to Fig 2 but lower power due to the weighted nature of our risk model for simulating the disease status. While the results described up to now pertain to simulation settings where the locus-specific *ϵ* and *c* are sampled independently for cases and controls (even when the average values are the same between the two groups), we also considered the setting in which *ϵ* and *c* are the same between cases and controls at each locus. This would occur when the two groups have been sequenced together through the exact same pipeline. As shown in S4 Fig, the power of New-SB and New-STB are always greater than or equal to the power of CG-S.

### UK10K data

The UK10K project [4] was funded by the Wellcome Trust Sanger Institute in 2010 to help investigators better understand the link between low-frequency and rare genetic changes and complex human diseases by applying NGS on 10,000 people in the United Kingdom (UK). We focused on the samples collected by the Severe Childhood Onset Obesity Project (SCOOP), all of whom have severe, early onset obesity (i.e., body mass index Standard Deviation Scores [22] > 3 and obesity onset before the age of 10 years). For controls, we utilized the population-based cohort collected in the TwinsUK study (randomly excluding one twin from each twinship) from the Department of Twin Research and Genetic Epidemiology at King’s College London. Both cases and controls are UK-based populations and part of the UK10K project. While the cases were whole-exome sequenced at average depth of 60×, the controls were whole-genome sequenced at average depth of 6×.

We used SAMtools to generate the pileup files from the BAM files and extracted read count data, filtering out reads that are PCR duplicates, that have mapping score < 30, that have improperly mapped mates, or that have *phred* base-quality scores < 30. We restricted our analysis to the consensus coding sequence gene sets [23] and further masked repeat regions, regions covered by monomorphic read alleles, and regions not covered by any reads, resulting in a total of ∼ 14 million loci exome wide. We recorded read count data for these loci such that, for example, a locus covered by 10 reads of allele A and 1 read of C was coded as A10C1. Read count datasets in this format are much more manageable than the BAM files; our formatted, zipped files required only 126 GB of disk space, compared to ∼ 14 TB for the BAM files. We obtained data in this format for 784 cases and 1,669 controls. We found that 87 cases had excessive read data that do not fit the binomial model (i.e., *Q* > 10) and we excluded these subjects (plus 1 additional case which is possibly in the same batch as the 87 cases) from further analysis; see S6 Text for more details. Thus the analysis described here was based on 696 cases and 1,669 controls.

We considered two versions for the weighted burden test, one including all variants and one including only variants that are annotated as “probably damaging” or “possibly damaging” by PolyPhen [24]. We applied our methods, New-SB and New-STB, to scan all genes for association with severe childhood onset obesity. We set *K*_max_ = 10,000,000, which is sufficient for detecting *p*-values that pass the exome-wide threshold that is on the order of 10^−6^. The analysis of damaging variants took a total of 1,713 hours on an IBM HS22 machine or equivalently 8.6 hours on 200 such machines in a computing cluster. We also applied the genotype calling method (CG-S) and the Derkach method (Dose-S) as described in Simulation Studies. Further, we analyzed the genotypes in the VCF files downloaded from the UK10K website. These genotypes were called by SAMtools, filtered by GATK VQSR, and imputed by Beagle [25], by the UK10K investigators with cases and controls being processed separately. We refer to this approach as CG-VCF.

We screened in a total of 474,508 loci, among which 465,967 (98.2%) loci passed our read-based QC procedure. The 465,967 loci span over 16,318 genes; 431,311 passed the threshold of (2*n*)^−1^ and 288,535 were estimated to be polymorphic. Considering damaging variants only, 238,753 loci were screened in and passed QC; 219,540 passed the threshold and 143,822 were estimated to be polymorphic. Note that the CG-VCF analysis was based on the same set of 465,967 loci, although some of them had been called monomorphic and were thus not included in the VCF files. As a result, the CG-VCF analysis included 167,980 loci, of which 79,271 were predicted as damaging.

**Fig 3:**
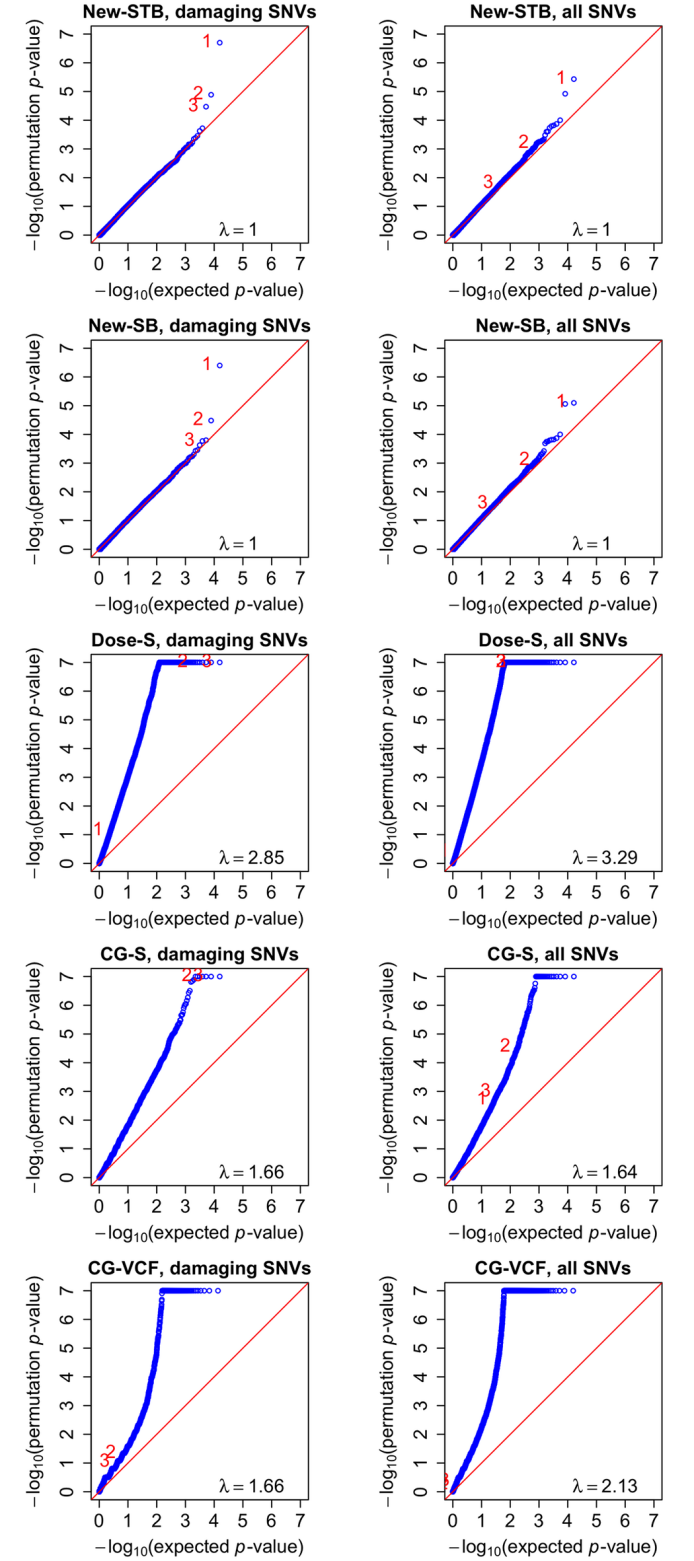
Quantile –quantile plots of −log_10_(*p*-values) for the weighted burden test using damaging SNVs only (left side) and all SNVs (right side) in the analysis of the UK10K data. The top three genes identified by New-STB using damaging variants only are marked as 1–3.

**Fig 4:**
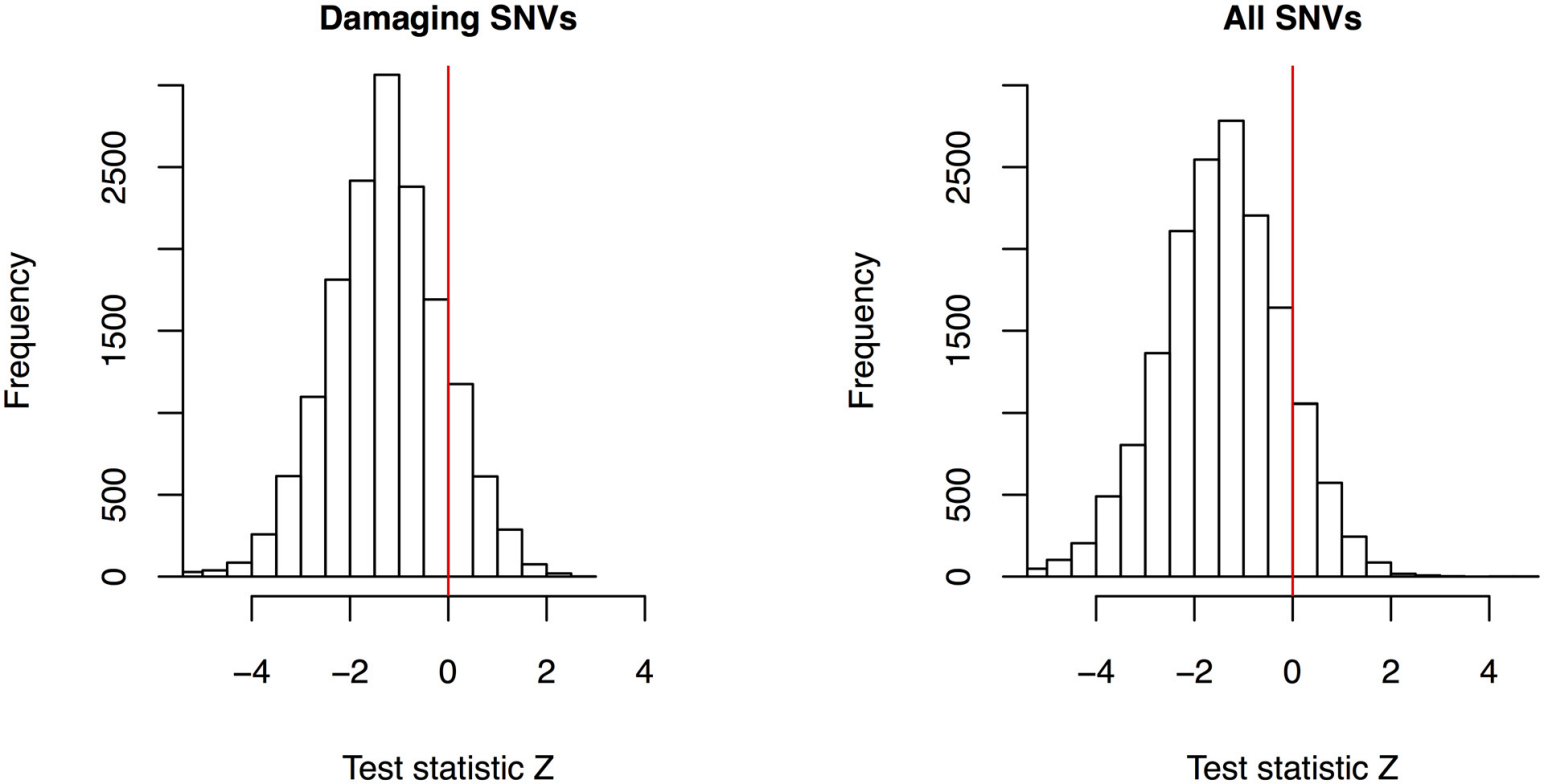
Distributions of the test statistic *Z* using damaging SNVs only (left side) and all SNVs (right side) in the analysis of the UK10K data. The left and right histograms are based on 15,659 and 16,318 genes, respectively.

The quantile-quantile plots are displayed in Fig 3. The observed *p*-values for New-STB and New-SB agree very well with the global null hypothesis of no association (genomic control *λ* = 1), except at the extreme right tails. By contrast, the observed *p*-values for Dose-S, CG-S, and CG-VCF show very early departures from the global null distribution, reflecting severe inflation of type I error. Fig 4 shows that the test statistics are negatively biased from zero, which explained the poor performance of Dose-S.

Among all *p*-values generated by our methods, the smallest one, 2.0 × 10^−7^, was obtained for gene *BTBD18* by New-STB using damaging variants only, and this *p*-value passed the exome-wide significance threshold of 3.1 × 10^−6^ (0.05/16,318) after Bonferroni correction. Looking into the raw read data on this gene, we found that among cases the WES resulted in extremely low depth (∼ 0.34×). (This kind of regions is not uncommon; indeed, 1.9% of all loci that were screened in have depth ≤1× in cases.) We found that at each of four loci (57512143, 57512745, 57513287, and 57513568 when mapped to the hg19 reference genome), there is a case individual covered by two reads and both are minor allele reads. These four suggestive minor allele homozygotes made large contributions to the score statistic and drove the gene-level association signal. As gene *BTBD18* has also been found to over-express in obese children elsewhere (NCBI GEO Profile ID: 64932244), it makes a plausible candidate for childhood onset obesity. Table 3 lists *BTBD18* and other top ten genes ranked by New-STB using damaging variants. Note that the standard genotype calling approach (CG-VCF) would have precluded *BTBD18* from association analysis due to the low depth data in cases. Using all SNVs, *BTBD18* was also ranked highest by New-STB, with the same four loci driving the association signal, but the *p*-value did not pass the exome-wide significance threshold because of the inclusion of other neutral variants.

**Table 3:** Top ten genes for childhood onset obesity identified by New-STB using damaging variants in the analysis of the UK10K data.

			New-STB		New-SB			Dose-S	CG-S	CG-VCF	
Gene	Chr	*L*	*M*_*st*_	*p* -value	*M*_*s*_	${\hat{M}}_{p}$	*p* -value	*p* -value	*p* -value	*M*	*p* -value
*BTBD18*	11	390	9	2.0 × 10^−7^	13	6.3	4.0 × 10^−7^	7.1 × 10^−2^	9.8 × 10^−1^	NA	NA
*OLFM1*	9	638	30	1.3 × 10^−5^	31	22.4	3.3 × 10^−5^	1.2 × 10^−13^	4.4 × 10^−8^	5	4.6 × 10^−2^
*UBR4*	1	5303	107	3.4 × 10^−5^	118	72.0	1.7 × 10^−4^	4.7 × 10^−16^	1.3 × 10^−10^	37	9.4 × 10^−2^
*HTR3C*	3	541	9	1.9 × 10^−4^	9	3.6	1.9 × 10^−4^	2.2 × 10^−2^	1.1 × 10^−2^	9	3.6 × 10^−3^
*GP6*	19	547	24	2.4 × 10^−4^	25	16.3	2.4 × 10^−4^	1.5 × 10^−2^	5.9 × 10^−4^	12	1.0 × 10^−4^
*PPARGC1B*	5	1141	36	3.5 × 10^−4^	38	19.5	3.5 × 10^−4^	8.8 × 10^−2^	1.7 × 10^−2^	20	1.5 × 10^−2^
*ISX*	22	272	6	4.1 × 10^−4^	8	5.2	2.1 × 10^−3^	1.9 × 10^−2^	2.7 × 10^−3^	6	1.6 × 10^−3^
*ZNF439*	19	863	15	4.5 × 10^−4^	16	13.0	3.8 × 10^−4^	2.3 × 10^−6^	5.7 × 10^−6^	7	6.1 × 10^−2^
*LMCD1*	3	506	14	5.0 × 10^−4^	14	7.7	5.0 × 10^−4^	3.4 × 10^−7^	8.7 × 10^−6^	6	4.7 × 10^−3^
*CLDN3*	7	305	24	6.8 × 10^−4^	24	12.8	6.8 × 10^−4^	4.6 × 10^−13^	5.5 × 10^−4^	4	3.8 × 10^−3^

Chr is the chromosome number. *L* is the total number of loci (base pair) in the gene. *M*_*s*_ is the number of loci that were screened in. *M*_*st*_ is the number of loci that were screened in and passed the threshold. ${\hat{M}}_{p}$ is the estimated number of SNVs. NA is not available.

## Discussion

We have presented a robust and efficient approach to association testing of rare variants that is based on analyzing raw sequencing reads directly, without calling genotypes. Our bootstrap procedure guarantees that the corresponding association tests have correct type I error under a wide range of sequencing differences between cases and controls. Our simulation studies showed that the proposed methods perform better than or as well as the genotype calling method in terms of power, when the latter shows no significant increase in type I error (e.g., when the average read depths and error rates are the same between cases and controls). These results can be understood by noting that converting reads into genotype data is a coarsening of the read data, which can result in information loss even when there is no differential error between cases and controls. These results suggest that, if the main goal is burden-based association testing (which is, in most cases, the goal of sequencing studies), then our proposed methods may be an attractive alternative to analyses based on called genotypes, even in studies where cases and controls have been “well-matched” for average depths or, further, have been sequenced together.

When applied to real data, our read-based procedure allows use of far more loci than methods based on calling genotypes, because we do not filter out variants covered by low depth of reads or called with low quality scores. For example, in analysis of the UK10K data, we only filtered out 1.8% of loci that were screened in; our final analysis included data from 465,967 loci. By contrast, the UK10K Statistics Group had to pare down to only 132,984 loci in order to achieve accurate type I error in the standard genotype calling approach, even though their analysis included almost 2,000 additional control participants from the Avon Longitudinal Study of Parents and Children (ALSPAC).

We have presented our methods in the context where all cases are from a single source and all controls are from another source. In practice, it is also common to use cases or controls from multiple sources, all from different platforms. The methods we have presented here can readily be extended to such scenarios by estimating a separate error rate for each data source, and then generating bootstrap datasets with the same source characteristics as the original data. We plan to implement this in future work.

When developing our methods, we made some simplifying assumptions. First, we assumed independence (i.e., no LD) across rare variants when generating bootstrap replicates. This is reasonable because rare variants typically do not exhibit strong LD with each other [26]. However, if strong LD occurs, it is possible to generate SNVs that have the same amount of LD as the original data by sampling haplotypes instead of single SNVs. The SNVs in the bootstrap sample can be placed in the same order (by allele frequency) as the original data.

Second, we assumed that base-calling errors are independent across loci. In reality, the base-calling errors might be correlated due to factors such as library preparation and sequence context. However, this assumption only affects the efficiency of our method, not its validity. We also assumed that the errors are symmetric, i.e., the probability of a read for the major allele being mis-called as the minor allele is the same as the probability of the minor allele being mis-called as the major allele. For analyzing rare variant data, this assumption has a negligible effect as rare variant homozygotes are extremely rare. Further, our methods estimate error rates directly from the read data, and thus ignored *phred* scores that characterize the base-calling quality and alignment scores that calibrate alignment quality. In our analysis of the UK10K data, we filtered out reads with alignment scores < 30 and *phred* scores < 30. We have shown in other work [27] that *phred* scores and low-score reads can provide additional information. It would be possible to include a model of the variability in error rates that is explained by base-calling and alignment quality scores in our current approach.

Finally, we do not account for confounders such as principal components for ancestry. In the UK10K data, all samples are UK-based Caucasians and are therefore not expected to have strong population stratification. It is also possible to extend our methods to allow confounders, by generating bootstrap replicates that have the same amount of confounding as the original data. We plan to describe such approaches in a subsequent report.

Our bootstrap procedure is parametric in the sense that its validity depends on correctly modeling the error and allele frequency distributions required to generate the bootstrap replicates. In addition, any added power that could be realized by relaxing assumptions like no LD across variants and independent error rates across loci would require special modification of our procedure. Further, we have assumed there are no confounding covariates; we plan to extend our approach to account for confounding covariates in future work. Finally, even with a sequential stopping rule, our bootstrap procedure may still be computationally intensive when the *p*-value to be estimated is very small. It may be possible to adopt a dynamic scheduling system so that nodes that are calculating a region having a large *p*-value would then shift their resources to regions where early bootstrap replicates suggest a small *p*-value.

We have focused on the burden test in this article. Because our score statistic may not have mean zero after screening, it is nontrivial to construct the sequence kernel association test (SKAT) [28]. A valid SKAT statistic requires the score statistic be properly centered; we are currently developing methods to center the score statistic within our bootstrap approach.

## Supporting Information

S1 Text Score statistic. (PDF)

S2 Text EM algorithm. (PDF)

S3 Text Proof for concavity of *pl*(*π*). (PDF)

S4 Text Empirical Bayes estimator of error rates. (PDF)

S5 Text Distributions for generating sequencing reads in simulation studies. (PDF)

S6 Text Details for excluding 88 UK10K case subjects. (PDF)

S1 Table Type I error of the unweighted burden test at the nominal significance level of 0.01. (PDF)

S2 Table Other simulation results for the unweighted burden test under the null hypothesis. (PDF)

S1 Fig Distributions of 2,730 locus-specific error rates from one replicate of the simulation studies. True is the error rate used in the simulation. MLE is the estimated error rate by the EM algorithm. EB is the empirical Bayes (EB) estimate. aEB is the adjusted EB estimate.
(TIF)

S2 Fig
${\hat{F}}_{s}$, $\hat{\phi }{\hat{F}}_{m}$, and $\left(1-\hat{\phi }\right){\hat{F}}_{p}$.*π* is the MAF. Each curve for ${\hat{F}}_{s}$ pertains to one replicate of the simulation studies and the curves for $\hat{\phi }{\hat{F}}_{m}$ and $\left(1-\hat{\phi }\right){\hat{F}}_{p}$ pertain to one bootstrap sample of that replicate. Green lines represent the threshold of (2*n*)^−1^.(TIFF)

S3 Fig Power of the unweighted burden test at the nominal significance level of 0.01. The title of each plot lists the average depths in cases and controls and then the average error rates in cases and controls. AR is the attributable risk per SNV. Each power estimate is based on 1,000 replicates. When there are differential average depths between cases and controls, CG-S and Dose-S have inflated type I error (S1 Table), so it is meaningless to compare their power with other methods.
(TIFF)

S4 Fig Power of the burden test at the nominal significance level of 0.01 when cases and controls have been sequenced together through the exact same pipeline. The title of each plot lists the average depths in cases and controls and then the average error rates in cases and controls. AR is the attributable risk per SNV. Each power estimate is based on 1,000 replicates.
(TIFF)

S5 Fig Distributions of locus-specific mean depth observed in the UK10K data (top panel) and generated in the simulation studies (bottom panel). We based on *Beta*(2.1, 4.1) and *Beta*(4.6, 4.8) to simulate locus-specific mean depths for cases and controls, respective, 2 were then re-scaled to achieve the average depths of 30× (bottom left) and 6× (bottom right).
(TIF)

S6 Fig Checking for UK10K case subjects with problematic data by raw read data. Case subjects 1 and 88 show typical patterns as observed among subjects 1–51 and 53–88. Subjects 89 and 94 show typical patterns as observed among subjects 89–784 and 52.
(TIF)

S7 Fig Checking for UK10K case subjects with problematic data by the *Q* value. The red vertical line separates the first 88 subjects and the remaining subjects.
(TIFF)
