# Supplementary material for: Testing Rare-Variant Association without Calling Genotypes Allows for Systematic Differences in Sequencing between Cases and Controls
Source: PLoS Genet. 2016 May 6;12(5):e1006040. doi: 10.1371/journal.pgen.1006040 (PMC4859496; doi:10.1371/journal.pgen.1006040)
Supplement: S1 Text — (PDF) [file pgen.1006040.s001.pdf]

S1 Text. Score statistic

The minor allele frequencies π_1 and π_0 for cases and controls, respectively, are re-parameterized as $\pi_{D_i} = e^{\alpha+\beta D_i}/(1+e^{\alpha+\beta D_i})$ for individual i. Denote $p_i = P_{\epsilon_{D_i}}(R|T, G = 0)+2e^{\alpha+\beta D_i}P_{\epsilon_{D_i}}(R|T, G = 1) + e^{2\alpha+2\beta D_i}P_{\epsilon_{D_i}}(R|T, G = 2)$ and $q_i = 2e^{\alpha+\beta D_i}P_{\epsilon_{D_i}}(R|T, G = 1) + 2e^{2\alpha+2\beta D_i}P_{\epsilon_{D_i}}(R|T, G = 2)$. The likelihood function (2) becomes $L_{\text{CC}}(\alpha, \beta, \epsilon_1, \epsilon_0) = \prod_{i=1}^{n} p_i/(1 + e^{\alpha+\beta D_i})^2$ and the score functions with respect to β and α are $S_\beta = \sum_{i=1}^{n} D_i \left\{q_i/p_i - 2e^{\alpha+\beta D_i}/(1 + e^{\alpha+\beta D_i})\right\}$ and $S_\alpha = \sum_{i=1}^{n} \left\{q_i/p_i - 2e^{\alpha+\beta D_i}/(1 + e^{\alpha+\beta D_i})\right\}$, respectively. Under the null hypothesis that $H_0 : \beta = 0$, the score functions S_β and S_α becomes $\sum_{i=1}^{n} D_i q_i/p_i - 2n_1 e^{\alpha}/(1 + e^{\alpha})$ and $\sum_{i=1}^{n} q_i/p_i - 2ne^{\alpha}/(1 + e^{\alpha})$, respectively. Then, we deduce that $S_\beta = S_\beta - (n_1/n)S_\alpha + (n_1/n)S_\alpha = \sum_{i=1}^{n}(D_i - n_1/n)q_i/p_i + (n_1/n)S_\alpha$. When the nuisance parameters α, ϵ_1, and ϵ_0 are substituted by their restricted MLEs, q_i/p_i is exactly $\widetilde{G}_i$ as defined below equation (3) and $S_\alpha = 0$ by the definition of restricted MLEs. Therefore, we have shown that the score statistic takes the form in equation (3).
