# Supplementary material for: Testing Rare-Variant Association without Calling Genotypes Allows for Systematic Differences in Sequencing between Cases and Controls
Source: PLoS Genet. 2016 May 6;12(5):e1006040. doi: 10.1371/journal.pgen.1006040 (PMC4859496; doi:10.1371/journal.pgen.1006040)
Supplement: S2 Text — (PDF) [file pgen.1006040.s002.pdf]

S2 Text. EM algorithm

The complete-data log-likelihood for a single locus is

$$l_c = \sum_{i=1}^{n} \sum_{g=0,1,2} I(G_i = g) \left[R_i \log e_g(\epsilon_{D_i}) + (T_i - R_i) \log \{1 - e_g(\epsilon_{D_i})\} + \log P_{\pi_0}(g) \right],$$

where $e_g(\epsilon_{D_i}) = \epsilon_{D_i}$, 0.5, and $1 - \epsilon_{D_i}$ when $g = 0$, 1, and 2, respectively. In the E-step, we evaluate $\mathrm{E}\{I(G_i = g | R_i, T_i, D_i)\}$, which can be shown to be

$$\omega_{ig} = \frac{P_{\epsilon_{D_i}}(R_i | T_i, G_i = g) P_{\pi_0}(G_i = g)}{\sum_{g'=0}^{2} P_{\epsilon_{D_i}}(R_i | T_i, G_i = g') P_{\pi_0}(G_i = g')}.$$

In the M-step, we maximize l_c with $I(G_i = g)$ replaced by ω_{ig}. Specifically, define $t_{0,\mathcal{D}_1} = \sum_{i \in \mathcal{D}_1} T_i \omega_{i0}$, $t_{0,\mathcal{D}_0} = \sum_{i \in \mathcal{D}_0} T_i \omega_{i0}$, $t_{2,\mathcal{D}_1} = \sum_{i \in \mathcal{D}_1} T_i \omega_{i2}$, $t_{2,\mathcal{D}_0} = \sum_{i \in \mathcal{D}_0} T_i \omega_{i2}$, $r_{0,\mathcal{D}_1} = \sum_{i \in \mathcal{D}_1} R_i \omega_{i0}$, $r_{0,\mathcal{D}_0} = \sum_{i \in \mathcal{D}_0} R_i \omega_{i0}$, $r_{2,\mathcal{D}_1} = \sum_{i \in \mathcal{D}_1} R_i \omega_{i2}$, $r_{2,\mathcal{D}_0} = \sum_{i \in \mathcal{D}_0} R_i \omega_{i2}$, $c_1 = \sum_{i=1}^{n} \omega_{i1}$, and $c_2 = \sum_{i=1}^{n} \omega_{i2}$, The minor allele frequency and the error rates are updated as

$$\pi_0 = \frac{c_1 + 2c_2}{2n}, \qquad \epsilon_1 = \frac{t_{2,\mathcal{D}_1} - r_{2,\mathcal{D}_1} + r_{0,\mathcal{D}_1}}{t_{0,\mathcal{D}_1} + t_{2,\mathcal{D}_1}}, \qquad \epsilon_0 = \frac{t_{2,\mathcal{D}_0} - r_{2,\mathcal{D}_0} + r_{0,\mathcal{D}_0}}{t_{0,\mathcal{D}_0} + t_{2,\mathcal{D}_0}}.$$
