# Supplementary material for: Testing Rare-Variant Association without Calling Genotypes Allows for Systematic Differences in Sequencing between Cases and Controls
Source: PLoS Genet. 2016 May 6;12(5):e1006040. doi: 10.1371/journal.pgen.1006040 (PMC4859496; doi:10.1371/journal.pgen.1006040)
Supplement: S3 Text — (PDF) [file pgen.1006040.s003.pdf]

S3 Text. Proof for concavity of $pl(\pi)$

First, we prove that, for fixed ϵ, the function $h(\pi) = \log\big\{\sum_{g=0,1,2} P_\epsilon(R|T,g)P_\pi(g)\big\}$ is concave. Under HWE, we write $h(\pi) = \log\big\{a_0(1-\pi)^2 + 2a_1\pi(1-\pi) + a_2\pi^2\big\}$, where $a_0 = \epsilon^R(1-\epsilon)^{T-R}$, $a_1 = 0.5^T$, and $a_2 = \epsilon^{T-R}(1-\epsilon)^R$. The second derivative of $h(\pi)$ is

$$h''(\pi) = -\frac{2\big\{(a_0 - 2a_1 + a_2)\pi + (a_1 - a_0)\big\}^2 + 2(a_1^2 - a_0a_2)}{\big\{a_0(1-\pi)^2 + 2a_1\pi(1-\pi) + a_2\pi^2\big\}^2}.$$

Because $a_0a_2 = \{\epsilon(1-\epsilon)\}^T \leq 0.25^T = a_1^2$, we obtain that $h''(\pi) \leq 0$ and thus $h(\pi)$ is a concave function of π.

Because the sum of concave functions are still concave, $\log L_{\mathrm{S}}(\pi,\epsilon)$ is concave in π for fixed ϵ. Because taking maximum of ϵ maintains the concavity [1], $pl(\pi)$ is also concave.

1. Boyd S, Vandenberghe L. Convex optimization. Cambridge University Press; 2004.
