# Supplementary material for: Testing Rare-Variant Association without Calling Genotypes Allows for Systematic Differences in Sequencing between Cases and Controls
Source: PLoS Genet. 2016 May 6;12(5):e1006040. doi: 10.1371/journal.pgen.1006040 (PMC4859496; doi:10.1371/journal.pgen.1006040)
Supplement: S4 Text — (PDF) [file pgen.1006040.s004.pdf]

S4 Text. Empirical Bayes estimator of error rates

Assume that ϵ_j follows a prior distribution $Beta(a, b)$ and the total number of errors across individuals, denoted by r_j, follows a binomial distribution $Bin(t_j, \epsilon_j)$. Under this model we can obtain the expected values of the (weighted) empirical first and second moments $m_1 = \sum_{j=1}^{M} \widetilde{\epsilon}_j t_j / \sum_{j=1}^{M} t_j$ and $m_2 = \sum_{j=1}^{M} \widetilde{\epsilon}_j^2 t_j / \sum_{j=1}^{M} t_j$, where m_1 and m_2 are weighted by the total number of reads t_j at locus j across all individuals. We estimate hyperparameters a and b using the method of moments, equating the empirical moments to their theoretical values. We find $\widehat{a} = B^{-1} m_1(m_1 - m_2)$ and $\widehat{b} = B^{-1}(1 - m_1)(m_1 - m_2)$, where $B = m_2 - m_1 + m_1(1 - m_1)(1 - M/\sum_{j=1}^{M} t_j)$.

The posterior distribution of ϵ_j given r_j and t_j is also a beta distribution $Beta(r_j + a, t_j - r_j + b)$. Thus, the empirical Bayes (EB) estimator is $\mathrm{E}(\epsilon_j | r_j) = w_j a/(a + b) + (1 - w_j) r_j/t_j$, where $w_j = (a + b)/(a + b + t_j)$. In calculating the EB estimator, we use the values of $\widehat{a}$ and $\widehat{b}$ obtained by the method of moments.
