# Supplementary material for: Testing Rare-Variant Association without Calling Genotypes Allows for Systematic Differences in Sequencing between Cases and Controls
Source: PLoS Genet. 2016 May 6;12(5):e1006040. doi: 10.1371/journal.pgen.1006040 (PMC4859496; doi:10.1371/journal.pgen.1006040)
Supplement: S5 Text — (PDF) [file pgen.1006040.s005.pdf]

S5 Text. Distributions for generating sequencing reads in simulation studies

When the average depth is $30\times$, we generated the locus-specific mean depth c using $Beta(2.1, 4.1)$, which was then re-scaled to achieve the mean of 30; given c, we generated the individual T's from $NB(c, 0.16)$. This mimicked the level of variability seen in the case data of UK10K. When average depth is $6\times$ or $10\times$, we generated c using $Beta(4.6, 4.8)$ and T using $NB(c, 0.30)$, which mimicked the control data of UK10K. S5 Fig displays the distributions of locus-specific mean depths observed in UK10K and those generated in our simulation studies, and confirms that the simulated data closely resemble the real data. To achieve average error rates of 0.02% and 0.016%, we used $Beta(1.75, 8755)$ and $Beta(4.58, 28648)$, respectively, as observed in UK10K cases and controls. To achieve the average error rates of 1% and 0.5%, we used $Beta(88, 8755)$ and $Beta(44, 8755)$, which we selected by using the second shape parameter from UK10K cases and adjusting the first shape parameter to give the desired mean.
