# Supplementary material for: Testing Rare-Variant Association without Calling Genotypes Allows for Systematic Differences in Sequencing between Cases and Controls
Source: PLoS Genet. 2016 May 6;12(5):e1006040. doi: 10.1371/journal.pgen.1006040 (PMC4859496; doi:10.1371/journal.pgen.1006040)
Supplement: S6 Text — (PDF) [file pgen.1006040.s006.pdf]

S6 Text. Details for excluding 88 UK10K case subjects

For QC at the individual level, we focused on a set of common variants ($\tilde{\pi} > 0.05$), which are more informative than rare variants. We ordered the 784 case subjects according to the list of BAM files that was provided by the UK10K. We first observed in S6 Fig that the plots of raw read data R/T for subjects 1–51 and 53–88 did not show three clean bands centered at 0, 0.5, and 1, as subjects 89–784 and 52 do. For subjects 1–51 and 53–88, the large number of R/T values lying between 0 and 0.5 suggest the data do not fit the binomial model (1). Indeed, S7 Fig confirmed that subjects 1–51 and 53–88 have an excessive number of loci having Qs greater than 10. These subjects warrant a close examination as to why they failed our QC. Because we feared the order of BAM files was informative of batch or some other important factor, we also excluded observation 52.
