# Supplementary material for: Testing Rare-Variant Association without Calling Genotypes Allows for Systematic Differences in Sequencing between Cases and Controls
Source: PLoS Genet. 2016 May 6;12(5):e1006040. doi: 10.1371/journal.pgen.1006040 (PMC4859496; doi:10.1371/journal.pgen.1006040)
Supplement: S1 Table — (PDF) [file pgen.1006040.s007.pdf]

S1 Table. Type I error of the unweighted burden test at the nominal significance level of 0.01

				**Known SNVs**				**Unknown SNVs**			
c_1	c_0	ϵ_1	ϵ_0	**New**	**CG**	**Dose**	**True**	**New-SB**	**New-STB**	**CG-S**	**Dose-S**
6×	6×	0.02%	0.02%	0.011	0.019	0.009	0.009	0.011	0.011	0.017	0.008
30×	6×	0.02%	0.02%	0.011	0.101	0.010	0.010	0.009	0.009	0.032	0.020
30×	30×	0.02%	0.02%	0.011	0.015	0.010	0.011	0.011	0.012	0.014	0.011
30×	6×	0.02%	0.016%	0.012	0.097	0.011	0.013	0.010	0.010	0.033	0.022
10×	10×	1%	1%	0.010	0.013	0.009	0.010	0.014	0.012	0.014	0.010
30×	10×	1%	1%	0.012	0.049	0.011	0.013	0.013	0.012	0.029	0.182
30×	30×	1%	1%	0.009	0.012	0.009	0.010	0.009	0.009	0.011	0.009
30×	10×	1%	0.5%	0.009	0.029	0.008	0.008	0.008	0.007	0.033	0.110

c_1 and c_0 are average depths in cases and controls, respectively. ϵ_1 and ϵ_0 are average error rates in cases and controls, respectively. Each entry is based on 10,000 replicates.
