# Supplementary material for: Testing Rare-Variant Association without Calling Genotypes Allows for Systematic Differences in Sequencing between Cases and Controls
Source: PLoS Genet. 2016 May 6;12(5):e1006040. doi: 10.1371/journal.pgen.1006040 (PMC4859496; doi:10.1371/journal.pgen.1006040)
Supplement: S2 Table — (PDF) [file pgen.1006040.s008.pdf]

S2 Table. Other simulation results for the unweighted burden test under the null hypothesis

				New		New-SB			New-STB	
c_1	c_0	ϵ_1	ϵ_0	Z	M_p	Z	M_s	$\widehat{M}_p$	Z	M_{st}
6×	6×	0.02%	0.02%	0.001	19.9	0.002	47.6	19.6	0.002	46.0
30×	6×	0.02%	0.02%	0.114	21.3	-0.550	35.0	21.4	-0.549	34.0
30×	30×	0.02%	0.02%	-0.007	22.6	-0.006	25.5	22.4	-0.006	25.1
30×	6×	0.02%	0.016%	0.122	21.4	-0.524	34.8	21.4	-0.530	34.0
10×	10×	1%	1%	-0.005	20.5	-0.002	162.0	20.3	0.002	63.1
30×	10×	1%	1%	0.020	21.4	-1.433	102.0	20.9	-0.511	38.6
30×	30×	1%	1%	-0.014	22.3	-0.009	55.3	21.9	-0.014	27.9
30×	10×	1%	0.5%	-0.003	21.7	-1.203	89.7	21.2	-0.367	36.0

c_1 and c_0 are average depths in cases and controls, respectively. ϵ_1 and ϵ_0 are average error rates in cases and controls, respectively. Z is the test statistic. M_p is the number of true SNVs. $\widehat{M}_p$ is the estimated number of SNVs. M_s is the number of loci that were screened in. M_{st} is the number of loci that were screened in and passed the threshold. Each entry is based on 10,000 replicates.
