# Supplementary figures and images for: Testing Rare-Variant Association without Calling Genotypes Allows for Systematic Differences in Sequencing between Cases and Controls
Source: PLoS Genet. 2016 May 6;12(5):e1006040. doi: 10.1371/journal.pgen.1006040 (PMC4859496; doi:10.1371/journal.pgen.1006040)

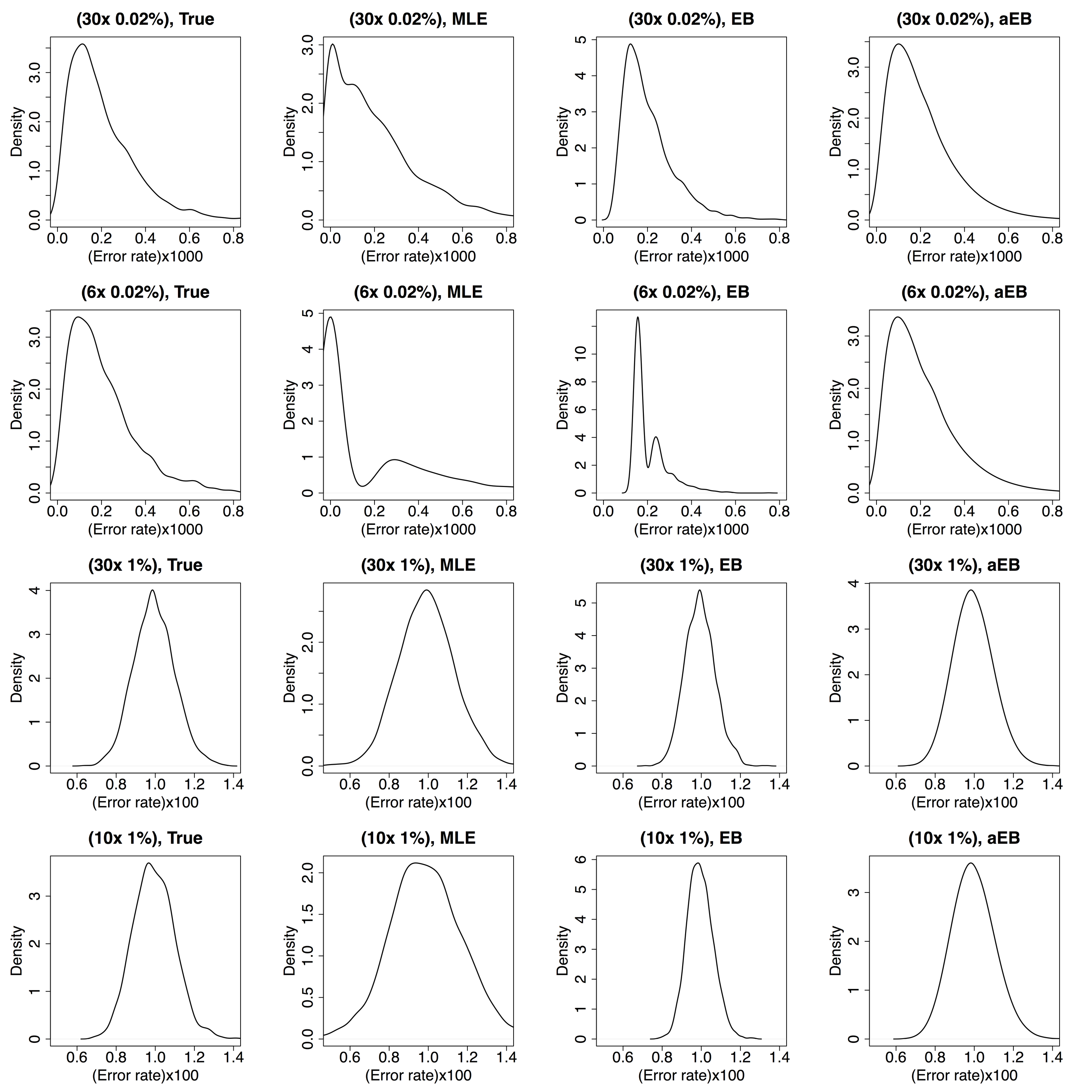

Supplement: S1 Fig — True is the error rate used in the simulation. MLE is the estimated error rate by the EM algorithm. EB is the empirical Bayes (EB) estimate. aEB is the adjusted EB estimate. (TIF) [file pgen.1006040.s009.tif]

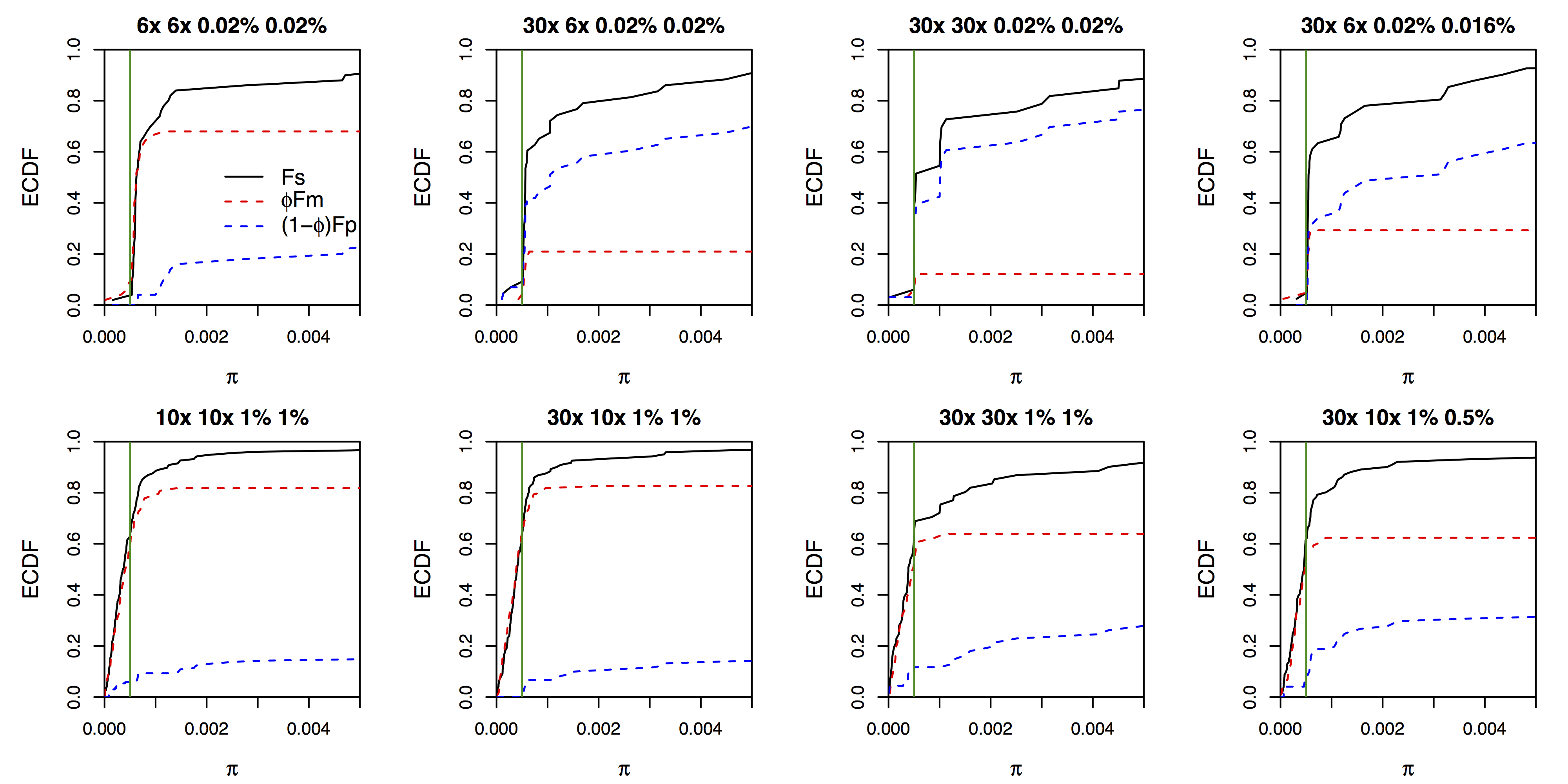

Supplement: S2 Fig — π is the MAF. Each curve for F^s pertains to one replicate of the simulation studies and the curves for ϕ^F^m and (1-ϕ^)F^p pertain to one bootstrap sample of that replicate. Green lines represent the threshold of (2n)−1. (TIFF) [file pgen.1006040.s010.tiff]

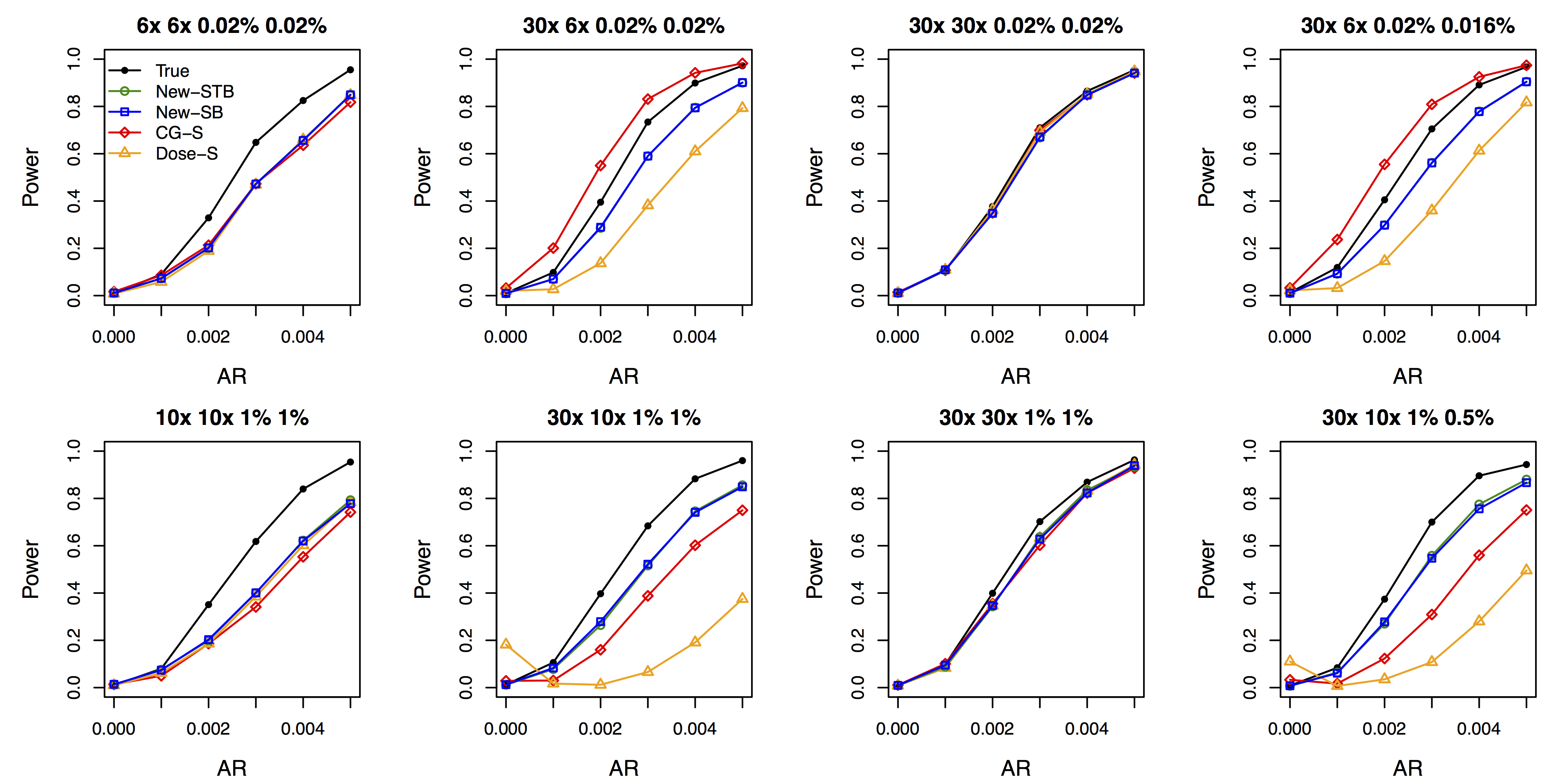

Supplement: S3 Fig — The title of each plot lists the average depths in cases and controls and then the average error rates in cases and controls. AR is the attributable risk per SNV. Each power estimate is based on 1,000 replicates. When there are differential average depths between cases and controls, CG-S and Dose-S have inflated type I error (S1 Table), so it is meaningless to compare their power with other methods. (TIFF) [file pgen.1006040.s011.tiff]

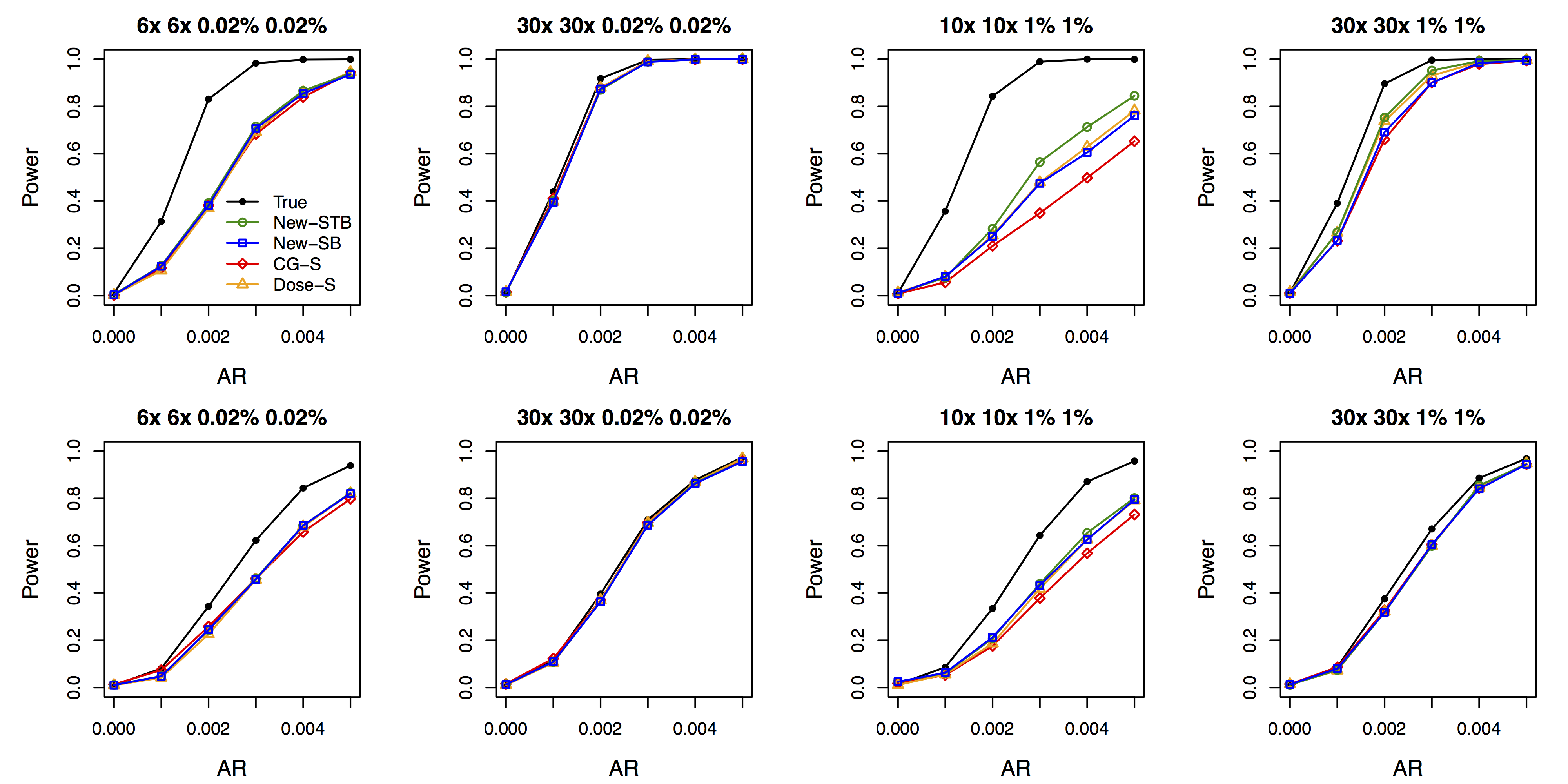

Supplement: S4 Fig — The title of each plot lists the average depths in cases and controls and then the average error rates in cases and controls. AR is the attributable risk per SNV. Each power estimate is based on 1,000 replicates. (TIFF) [file pgen.1006040.s012.tiff]

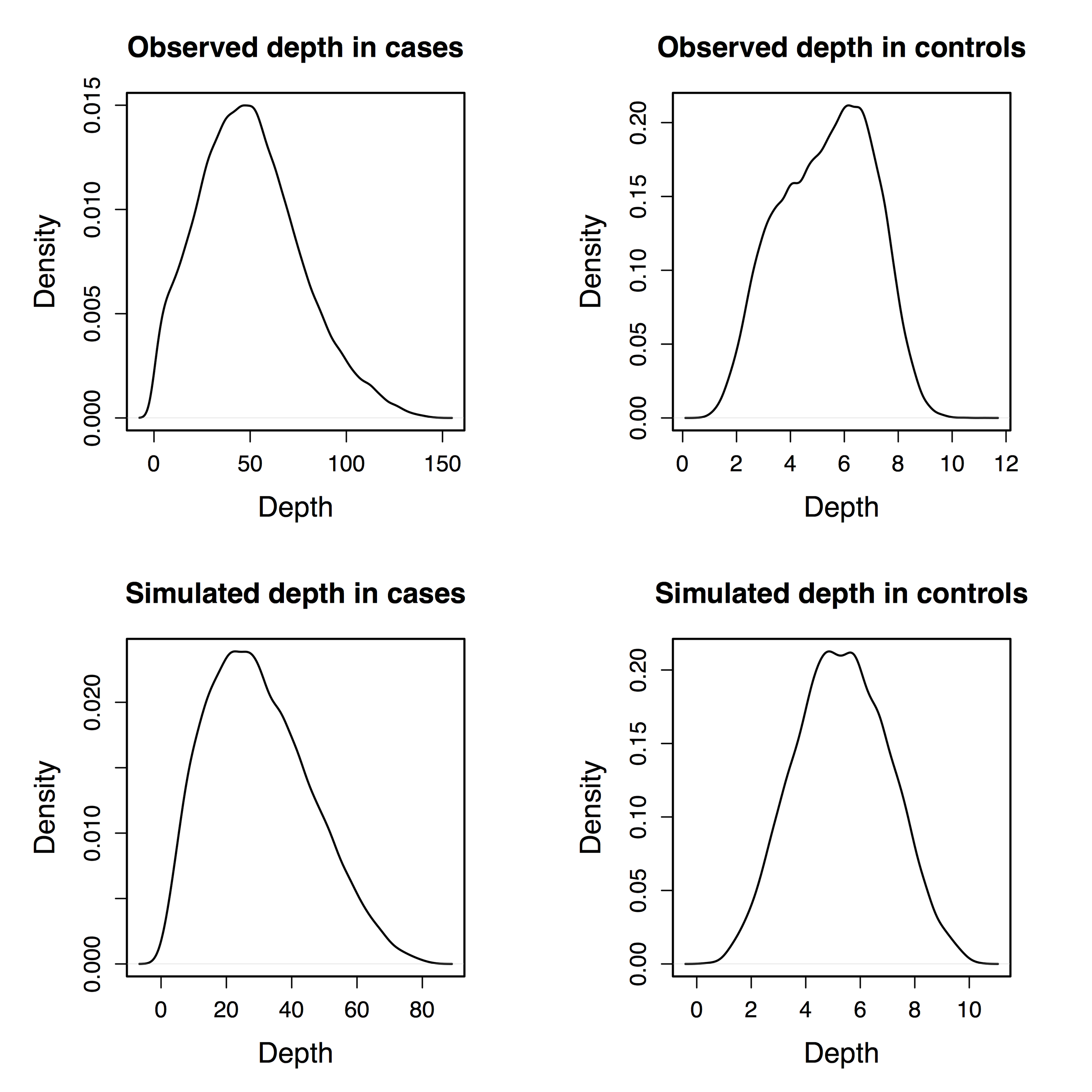

Supplement: S5 Fig — We based on Beta(2.1, 4.1) and Beta(4.6, 4.8) to simulate locus-specific mean depths for cases and controls, respective, 2 were then re-scaled to achieve the average depths of 30× (bottom left) and 6× (bottom right). (TIF) [file pgen.1006040.s013.tif]

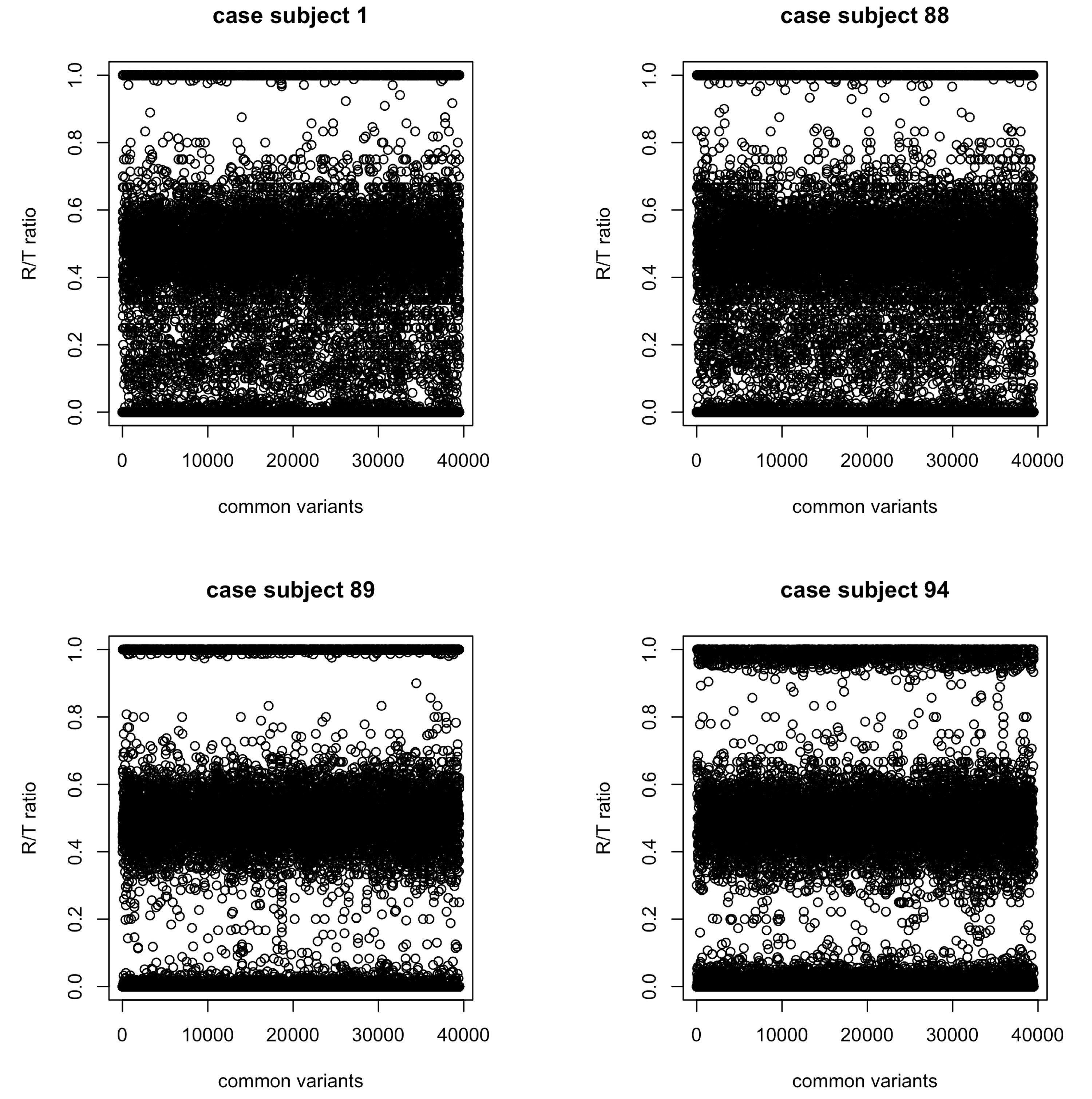

Supplement: S6 Fig — Case subjects 1 and 88 show typical patterns as observed among subjects 1–51 and 53–88. Subjects 89 and 94 show typical patterns as observed among subjects 89–784 and 52. (TIF) [file pgen.1006040.s014.tif]

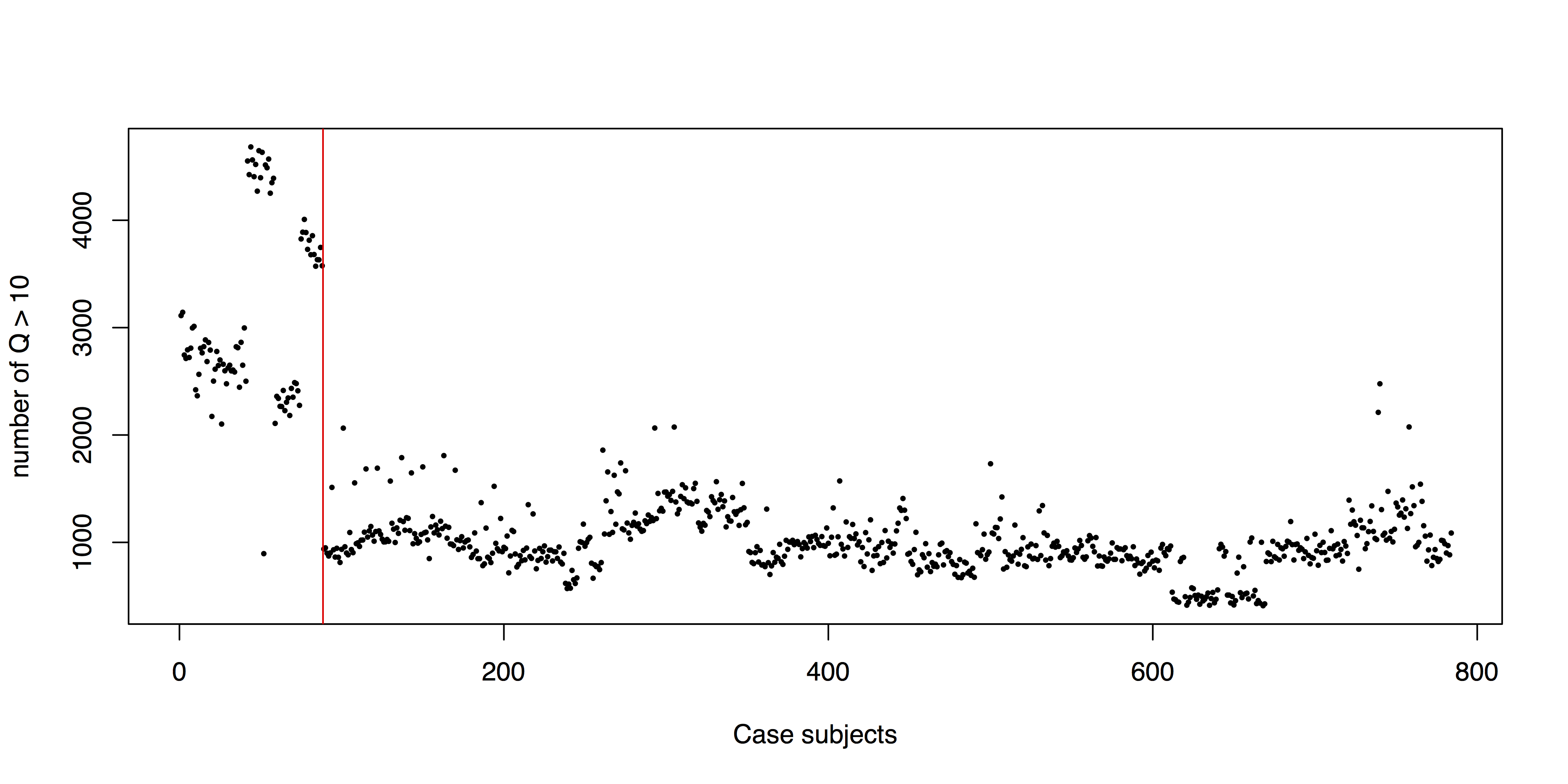

Supplement: S7 Fig — The red vertical line separates the first 88 subjects and the remaining subjects. (TIFF) [file pgen.1006040.s015.tiff]
